# The impact of temporal distribution on fear extinction learning

**DOI:** 10.1016/j.ijchp.2024.100536

**Published:** 2025-01-11

**Authors:** Yuanbo Ma, Dzheylyan Kyuchukova, Fujia Jiao, Giorgi Batsikadze, Michael A. Nitsche, Fatemeh Yavari

**Affiliations:** a Department of Psychology and Neurosciences, Leibniz Research Centre for Working Environment and Human Factors, Dortmund, Germany; b Department of Psychology, Ruhr University Bochum, Bochum, Germany; c Department of Psychotherapy and Systems Neuroscience, Justus Liebig University of Giessen, Germany; d Key Laboratory of Exercise and Health Sciences of Ministry of Education, Shanghai University of Sport, Shanghai, China; e Department of Neurology and Center for Translational Neuro and Behavioral Sciences (C-TNBS), Essen University Hospital, University of Duisburg-Essen, Hufelandstraße 55, Essen 45147, Germany; f Bielefeld University, University Hospital OWL, Protestant Hospital of Bethel Foundation, University Clinic of Psychiatry and Psychotherapy, Bielefeld, Germany; g German Center for Mental Health (DZPG), Bochum, Germany

**Keywords:** Fear conditioning, Fear extinction, Massed training, Spaced training, Temporal distribution, Skin conduction response, Extinction retention

## Abstract

Fear extinction is the foundation of exposure therapy for anxiety and phobias. However, the stability of extinction memory diminishes over time, coinciding with fear recovery. To augment long-term extinction retention, the temporal distribution of extinction learning sessions is critical. This study investigated the effects of massed and spaced training (with short and long intervals) on extinction retention compared to a classic protocol. 120 healthy participants were recruited and randomly divided to massed training, spaced training with 20-minutes or 3-hours intervals, and a control group. The control group completed half the number of extinction trials compared to the other groups. The fear conditioning/extinction paradigm consisted of three consecutive days of fear acquisition, extinction, and recall, followed by a second recall one week later. Skin conductance response (SCR) and self-rating questionnaires (ratings of valence, arousal, and fear) were recorded and analyzed using mixed model ANOVAs. The results revealed that during the extinction phase, both massed and spaced protocols showed significantly lower SCRs compared to the control group, with massed training resulting in the largest effects. In the second recall, only the massed extinction group showed no significant difference in SCRs between threat and safety cues. The self-report assessments indicated that the massed extinction group showed furthermore lower arousal than the control group in the first recall. These results suggest that both massed and spaced training promote fear extinction learning, but only massed training improves long-term extinction retention. This study highlights the impact of the temporal distribution and trial number of extinction learning on extinction retention, offering insights for future research on improving fear extinction efficacy.

## Introduction

Fear extinction, a fundamental cornerstone of exposure therapy (Hermans, Craske, Mineka, & Lovibond, 2006), plays a central role in treating anxiety and phobias (Graham & Milad, 2011; Stein & Matsunaga, 2006). It involves repeated exposure to a fearful condition in the absence of actual threat, promoting the formation of extinction memory and inhibiting the consolidation of fear memory (Myers & Davis, 2007). However, the temporal attenuation of extinction memory and the re-occurrence of fear memory pose challenges to the effectiveness of fear extinction (Lonsdorf, Merz, & Fullana, 2019). Enhancing the consolidation of extinction memory could serve as a crucial factor for facilitating the treatment of anxiety and phobias (Bouchet et al., 2017; Pace-Schott, Germain, & Milad, 2015). One aspect to be explored for enhancing extinction efficacy is the timing and distribution of extinction training sessions.

Extensive evidence supports the contribution of spaced training for the induction of a more persistent long-term memory (Cecilio-Fernandes, Cnossen, Jaarsma, & Tio, 2018; Josselyn et al., 2001; San Martin, Rela, Gelb, & Pagani, 2017). Spaced training, i.e. distributing learning sessions over time with specific intervals between them, has been shown to enhance learning outcomes and long-term memory retention (Bakkour et al., 2018; Glas, Hübener, Bonhoeffer, & Goltstein, 2021). Conversely, massed training involves intensive, uninterrupted practice in a single session. Spaced training is typically seen as more effective for long-term memory retention across various forms of human learning, such as vocabulary learning, fact and concept learning, skill and motor learning (Carpenter, Cepeda, Rohrer, Kang, & Pashler, 2012; Donovan & Radosevich, 1999; Shea, Lai, Black, & Park, 2000; Smolen, Zhang, & Byrne, 2016). The study-phase retrieval theory relates the enhancement of reactivation of a memory trace after each interval to memory reinforcement and is considered as one of the key concepts explaining the superiority of spaced training (Smolen et al., 2016). Not all available studies however support this concept. For example, in a study conducted in children, though four blocks of spaced training -with an interval of 2.5 min- were superior for enhancing performance accuracy in a word recognition task compared to massed training, the advantage of spaced training was not observed in a word spelling task (Wegener et al., 2022). Moreover, in an animal study, rats with massed training demonstrated superior performance in a hippocampus-dependent location memory task compared to spaced training with an interval of 30 min. In contrast, spaced training showed better performance than massed training in a dorsolateral striatum-dependent response task (Wingard, Goodman, Leong, & Packard, 2015). These findings underscore the influence of both, the training protocol and task type on the effects of spaced training on performance. In addition, evidence indicates the dependency of the efficiency of spaced training on the interval length, with intervals generally favoring memory retention within specific time windows, such that excessive time intervals may not yield correspondingly better benefits (Cepeda, Pashler, Vul, Wixted, & Rohrer, 2006; Kornmeier, Sosic-Vasic, & Joos, 2022). Here it has been shown that training with a 2-day interval was more effective for acquiring and retaining arthroscopic skills for 3 months compared to training with 1-day or 1-week intervals (Yang et al., 2023). For vocabulary learning and visual acuity tasks, a 12-hour spacing interval showed superior long-term memory retention and visual acuity over four weeks, compared to a 24-hour spacing interval (Kornmeier, Spitzer, & Sosic-Vasic, 2014). These studies indicate that timing of learning, such as spacing, plays a role for memory formation, but the respective parameter space is wide, has not been systematically explored and may differ between task types.

In the fear extinction field, both massed and spaced training enhance the efficiency of extinction (Gerhard & Meyer, 2021; Laborda, Polack, Miguez, & Miller, 2014). Adolescent male mice demonstrated fear extinction learning in both, massed training and spaced training conditions, the latter with two- or four-day intervals. Short- and long-term extinction retention were observed in all three training schedules relative to a no extinction group (Gerhard & Meyer, 2021). However, whether spaced or massed training is more effective for fear extinction learning is currently unclear (Jiang et al., 2016, 2019). In an animal study, two sessions of extinction training with a 7-day interval showed no superior effect on fear extinction learning compared to two sessions with a 24-hour interval. However, the 7-day spaced extinction sessions were more effective in maintaining extinction memory after 28 days (Tapias-Espinosa, Kádár, & Segura-Torres, 2018). In a study in humans, participants with public-speaking anxiety showed immediate fear reduction after exposure treatment in massed, four uniformly-spaced sessions (5, 5, 5 days interval), and four expanded-space sessions (1, 4, and 10 days interval) schedules, with the massed group producing the largest fear reduction, whereas in the 1-month follow-up, only the two spaced schedules did not show fear return (Tsao & Craske, 2000). In another human study, two groups of spider-phobic people were assessed after a spider exposure task with either a massed or spaced exposure schedule (four exposure trials within one day or distributed within one week). In that study, the massed exposure group showed superior decreases in self-reported fear, heart rate, and danger ratings. However, only the spaced exposure group did not show return of fear towards a novel spider after one month (Rowe & Craske, 1998). Meanwhile, recent clinical research found that both intensified (6 sessions completed over 2 weeks) and spaced exposure therapy (1 session per week for 6 weeks) reduced symptom severity and disability in patients with severe anxiety disorders, with these benefits persisting for six months post-treatment. However, the intensified exposure therapy showed faster symptom reduction, lower dropout rates, and better improvements in quality of life compared to spaced exposure therapy (Pittig et al., 2021). These findings suggest that massed training has superior immediate effects on memory retention. However, it remains inconclusive whether spaced or massed training results in more stable long-term effects.

With respect to the physiological foundation of learning and memory formation, it has been shown that the formation of memory is critically dependent on synaptic plasticity (Bailey, Kandel, & Harris, 2015; Kim & Jung, 2006; Rodrigues, Schafe, & LeDoux, 2004). Long-term potentiation (LTP) is the persistent enhancement of synaptic strength following a specific stimulus, a form of synaptic plasticity that has been widely studied as a potential mechanism for learning and memory formation (Nicoll, 2017; Pastalkova et al., 2006). Evidence suggests that inducing LTP in the medial prefrontal cortex (mPFC) during fear extinction is relevant to consolidate new safety memories, suppressing the fear response and maintaining extinction memory over time (Herry & Garcia, 2002; Herry, Vouimba, & Garcia, 1999). One crucial aspect of this process is late phase LTP (L-LTP), which involves the sustained strengthening of synapses for more than 3 h after stimulation (Huang, Pittenger, & Kandel, 2004; Reymann & Frey, 2007). This process necessitates gene expression and protein synthesis to induce alterations of synaptic strength, which is a likely foundation for prolonged effects of extinction learning (Costa-Mattioli, Sossin, Klann, & Sonenberg, 2009; Huang et al., 2004). Evidence suggests that this process can be induced by spaced training within specific intervals (Scharf et al., 2002). The specific intervals used in physiological studies are usually however shorter than those used in the behavioral studies mentioned above (Reymann & Frey, 2007). This suggests that the precise timing of training sessions is a critical factor influencing the effectiveness of memory retention, and may open a window for new timing intervals to enhance the efficacy of memory formation, and retention based on physiological grounds. It has been shown that spaced training with a 5-minute interval in mice induces larger LTP compared to massed training, and promotes long-term fear memory by maintaining L-LTP, which depended on protein synthesis (Scharf et al., 2002). Similarly, it has been shown in humans that repetitive transcranial DC stimulation, which induces LTP-like plasticity, at short intervals (3 or 20 min) induces L-LTP-like plasticity in the motor cortex for up to 24 h, compared to no or longer intervention intervals (3 or 24 h) (Agboada, Mosayebi-Samani, Kuo, & Nitsche, 2020; Monte-Silva et al., 2013). Thus, short interval spaced extinction training may facilitate the emergence of late LTP and improve long-term memory formation.

However, knowledge about the impact of spaced training sessions and the effect of specific time intervals between sessions on the efficacy of fear extinction learning in humans is limited. The objective of the present study is to investigate the impact of massed and spaced extinction training sessions with different intervals, as compared to a conventional extinction protocol, on extinction learning and retention. Based on previous evidence, we hypothesized that both massed training and spaced extinction sessions facilitate fear extinction learning compared to traditional training. Furthermore, we aimed to explore the efficacy of specific intervention intervals based on L-LTP mechanisms, which has not been extensively studied in connection with fear extinction. Specifically, we postulated that a short interval between spaced extinction sessions would prolong the preservation of extinction memory caused by an L-LTP-like effect.

## Materials and methods

### Participants

We recruited 120 healthy non-smoking participants, aged between18 and 40 years (24.53 ± 3.75, 64 females), through online advertisements. All participants were determined to be right-handed according to the Edinburgh Handedness Inventory (Oldfield, 1971). Participants were native German speakers or had a minimum proficiency level of B1 knowledge of the German language. Participants were excluded from the study if they met any of the following criteria: a history or evidence of neurological disease, any psychiatric condition or mental illness, pregnancy, smoking, alcohol or drug abuse, or use of CNS-acting medication, and participation in a fear conditioning/extinction learning paradigm prior to this study. Based on our previous physiological studies, we aimed to enhance long-term memory by short interval spaced training via inducing L-LTP-like processes, and contrasted these protocols with massed, and conventional extinction (Agboada et al., 2020; Monte-Silva et al., 2013). Participants were randomly assigned to one of four groups: group 1, massed extinction; group 2, 20-minutes interval between extinction sessions; group 3, 3-hours interval between extinction sessions; group 4, classic extinction protocol (Ma, Jiao, Batsikadze, Yavari, & Nitsche, 2024). For a medium effect size of *f* = 0.15 (set based on theoretical grounds, since closely related experiments providing specific data were not available), an α error of 0.05 and a power of 0.8, the estimated total sample size was 23 per group. To compensate for potential dropouts and outliers, we enrolled 30 participants per group. Three participants were excluded because they did not show successful fear acquisition (the average skin conductance response to the threat cue was not larger than the one to the safety cue during acquisition) (Asthana et al., 2013). Demographic information of all participants is shown in Table 1. The study conformed to the Declaration of Helsinki and was approved by the local Ethics Committee. All participants gave written informed consent prior to study participation and were financially compensated for participation.

**Table 1:** Demographic information, psychological evaluations, and US ratings are displayed. Group comparisons were conducted via one-way ANOVAs, with the exception of gender differences, which were analyzed via a Chi-square test.

	Massed	20-minutes	3-hours	Control	*df*	*F*	*p value*
Age (years)	24.00 (3.24)	23.93 (3.02)	25.80 (4.91)	24.33 (3.48)	3	1.607	0.192
Gender (M/F)	12/17	13/15	13/17	15/15	3	NA	0.917
Education (years)	16.16 (2.30)	16.45 (2.53)	16.56 (2.78)	15.58 (2.54)	3	0.880	0.454
US Intensity (mA)	23.23 (15.26)	32.40 (29.29)	20.66 (15.84)	20.10 (17.75)	3	2.273	0.084
DASS21-Depression	3.32 (2.71)	2.73 (2.05)	3.88 (3.66)	2.34 (2.02)	3	1.732	0.165
DASS21-Anxiety	2.79 (2.50)	2.65 (2.71)	1.69 (2.46)	3.10 (2.37)	3	1.576	0.200
DASS21-Stress	6.11 (4.66)	4.04 (3.56)	5.08 (3.45)	5.07 (3.73)	3	1.270	0.289
ASI-3-Physical Concerns	4.29 (3.73)	6.69 (5.58)	5.08 (4.24)	6.72 (4.61)	3	1.944	0.127
ASI-3-Cognitive Concerns	5.43 (4.73)	6.23 (4.55)	6.08 (4.72)	6.69 (4.96)	3	0.342	0.795
ASI-3-Social Concerns	7.50 (4.61)	7.62 (4.89)	8.96 (5.53)	8.48 (5.04)	3	0.523	0.667
Rating of the last US	7.83 (0.81)	7.79 (0.69)	8.07 (0.69)	7.60 (1.43)	3	1.200	0.313
US likelihood after CS+	0.66 (0.13)	0.63 (0.12)	0.61 (0.15)	0.59 (0.14)	3	1.577	0.199
US likelihood after CS-	0.00 (0.00)	0.01 (0.01)	0.00 (0.00)	0.01 (0.02)	3	0.718	0.543

*M* = Male; *F* = Female; US intensity refers to the stimulation level in milliamperes (mA); DASS21 = Depression Anxiety Stress Scale 21; ASI-3 = Anxiety Sensitivity Index 3; The last US rating refers to intensity ratings of the last unconditioned stimulus in the acquisition phase; US likelihood after CS+ and CS- refers to the likelihood assessments of US occurrence after presentation of each of the CSs during acquisition; values are presented as mean (standard deviation). “NA” indicates not applicable. Gender was analyzed with a chi-square test, and the chi square value was 0.509. No significant differences between groups for any measure were observed.

### Fear conditioning paradigm

**Fig. 1:**
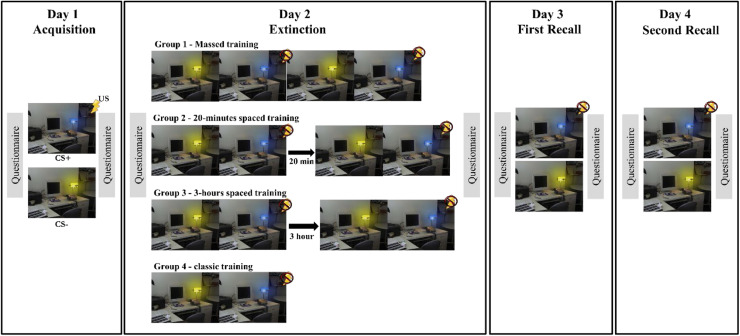
The fear conditioning paradigm design. On day 1, all participants received the fear acquisition protocol (16 CS+; 16 CS-; 10 US). On day 2, the respective groups received massed training, 20-minutes spaced training, 3-hours spaced training (all with 16 CS+; 16 CS-; no US), or the control protocol (8 CS+; 8 CS-; no US). On days 3 and 4, all participants performed the first and second recall sessions (8 CS+; 8 CS-; no US). The second recall was conducted one week after the extinction phase. CS+: reinforced conditioned stimulus, blue light; CS-: not reinforced conditioned stimulus, yellow light; US: unconditioned stimulus; Questionnaire: Prior to each phase, the Positive and Negative Affect Scale was completed by the participants. Following each phase, valence, arousal, and fear ratings were assessed.

The fear conditioning paradigm which was employed in this study was initially introduced by Milad and co-workers (Milad et al., 2007). It was conducted over four phases (Fig. 1): acquisition, extinction, first recall, and second recall. The first three phases were conducted over three successive days (approximately 24-hour intervals), and the second recall was administered one week after the extinction phase. Throughout the study, the context was kept constant as a picture of a desk lamp next to a computer on a table. Two types of conditioned stimuli (CS) were distinguished by the color of the lamp's light (CS+: blue, CS-: yellow). The unconditioned stimulus (US) was an electric pulse applied to the tip of the right index finger through an electrode. The US was exclusively presented following the CS+, and the CS- was never followed by the US.

The acquisition phase included 16 CS- and 16 CS+ trials. 10 CS+ trials were followed by the US (reinforcement rate of 62.5 %). Each trial consisted of 1 s of isolated context presentation, followed by 12 s of CS presentation. In reinforced trials, a 100 ms aversive pulse was administered as US, comprising four consecutive 500 µs current pulses applied with a 33 ms interval. The US was applied 11.9 s after CS onset, therefore co-terminating with the CS (Batsikadze et al., 2022). Between trials, a black background with a white cross in the middle was displayed (the intertrial interval, ITI, was randomized between 23 and 26 s). The trial duration and ITI remained consistent in all following phases. The duration of the acquisition phase was 20 min.

In the extinction and recall phases, no US was paired with any of the CS+ trials. In group 1, for massed extinction, 32 trials (16 CS+/16 CS-) were presented consecutively without interruption. In group 2 and group 3, the spaced extinction protocols, the 32 trials were divided into two sessions of 16 trials each, with a 20-minutes interval between sessions in group 2 and a 3-hours interval in group 3. In group 4, the control condition, 16 trials (8 CS+/8 CS-) were presented without interruption, a protocol shown to effectively establish fear extinction in our previous study (Ma et al., 2024). The extinction phase lasted 20 min for the first three groups and 10 min for group 4.

In both recall phases - day 3 and one week after extinction learning - all groups were exposed to 16 trials (8 CS+/8 CS-), and each phase lasted for 10 min.

Trial orders within each phase were organized in a pseudorandom manner, following four specific requirements: (i) during the acquisition phase, the first and the two last CS+ trials included the US; (ii) in each phase, no more than two consecutive trials were of the same type; (iii) the order of trials was the same for all participants; (iv) the number of each cue type was the same throughout the first and second half of each phase.

**Skin conductance response (SCR):** SCR was recorded using the Biopac system (MP160, EDA100c-MRI module, BIOPAC Systems Inc., Goleta, CA) throughout the entire task. The sample rate was 1 kHz, and the gain was 10 μS/V. The data were band-pass filtered (0.5–10 Hz) to avoid effects of high-frequency noise and low-frequency drifts. Two disposable standard snap electrodes (EDA BIOPAC EL509, BIOPAC Systems Inc., Goleta, CA) were placed on the thenar and hypothenar eminences of the non-dominant hand and participants were asked to avoid excessive movement throughout the task.

**Questionnaires:** Participants filled in two validated questionnaires (Depression Anxiety Stress Scale 21 -DASS-21, and Anxiety Sensitivity Index 3 -ASI-3) before the study to assess their psychological state. These questionnaires have good reliability for assessing depression, anxiety, and stress (Gámez et al., 2014; Lovibond & Lovibond, 1995; Peterson & Heilbronner, 1987). The DASS-21 is composed of three distinct dimensions—depression, anxiety, and stress—each assessed by 7 items, resulting in a 21-item evaluation. Participants rated each item on a four-point Likert scale, ranging from 0 (did not apply to me at all) to 3 (applied to me very much). The ASI-3 is structured into physical, cognitive, and social subscales, each with 6 items, resulting in 18 items. Participants rated each item on a five-point Likert scale, ranging from 0 (very little) to 4 (very much).

Prior to each phase, participants filled in the Positive and Negative Affect Scale (PANAS) to evaluate their current emotional state (Crawford & Henry, 2004). The scores of positive and negative affect were independently calculated, with each affect including of 10 items. Participants rated each item on a five-point Likert scale, ranging from 1 (very slightly) to 5 (extremely).

Following fear acquisition training, participants were asked to estimate the intensity of the last US on a nine-point Likert scale ranging from 0 (not unpleasant) to 9 (very unpleasant). In addition, the likelihood of the US occurrence after CS+ and CS- presentation was assessed after fear acquisition with a scale ranging from 0 to 100 %.

After each phase, participants were required to estimate valence, arousal and fear associated with the CS+ and CS-. Valence was rated on a five-point Likert scale ranging from one (very pleasant) to five (very unpleasant), and arousal and fear were assessed using visual analogue scales rating from 0 (calm and relaxed/not afraid at all) to 100 % (very excited/very afraid), respectively.

### Procedure

The study was conducted in a dimly lit and soundproof laboratory with a temperature which was maintained in the range of 22 to 24 °C. At the beginning of each phase, participants were seated in a comfortable chair in front of a computer screen (size 43.2 × 28.8 cm), and the basic experimental procedure was explained to them. Then, electrodes for SCR recording, and the US application were connected. The individualized US intensity was measured on the first day and pulses were applied using a constant current stimulator (DS7A, Digitimer Ltd., London, UK, maximum current output 100 mA). Participants were asked to report their perception of the applied pulse on a 9-point Likert scale (ranging from 0, “no or very slightly” to 9, “painful”). The process continued until a level of 8 “highly uncomfortable, but not painful” was reached. This measured intensity remained constant throughout the entire study.

At the initiation of the fear acquisition training, participants were instructed to note a potential association between the light color and the subsequent electrical pulse on their finger. At the onset of the following phases, participants were informed that any associations they identified on the first day would persist. The experimental paradigm was designed using Presentation software (version 23.0, Neurobehavioral System Inc., Berkeley, CA).

## Calculations and statistics

The SCR data were processed using Matlab software (Release 2021b, The MathWorks Inc., Natick, MA) with a semi-automated peak detection algorithm (Otto et al., 2023). To detect responses, a minimum amplitude of 0.01 μS and a minimum rise time of 0.5 s were defined (Boucsein et al., 2012). The SCR amplitudes were defined as the maximum trough-to-peak variation within the time window of 1 to 12.5 s following CS onset. The raw data underwent logarithmic transformation (after adding a constant of 1) to normalize the distribution (Keene, 1995; Lonsdorf et al., 2017).

The statistical analyses were performed using IBM SPSS Statistics software (IBM Corp., IBM SPSS Statistics for Windows, Version 29.0. Armonk, NY, United States). Demographics and psychological assessments (DASS-21, ASI-3) were evaluated using chi-square tests and one-way ANOVAs. PANAS data were analyzed with a mixed model ANOVA, with *Phase* (1, before acquisition; 2, before extinction; 3, before first recall; 4, before second recall) and *Type* (positive and negative) as within-subject factors, and *Group* (1, Massed; 2, 20-minutes; 3, 3-hours; 4, control) as the between-subject factor. For all ANOVA analyses, a Greenhouse-Geisser correction was applied if sphericity was violated as shown by Mauchly's sphericity test. Post-hoc Fisher's Least Significant Difference (LSD) tests were performed in case of significant ANOVA results, with statistical significance set at p < 0.05.

A mixed model ANOVA was conducted to analyze SCR data for each phase separately, with Group (4 groups) as the between-subject factor. In the acquisition phase, *Stimulus* (CS+, CS-) and *Trial* (1 to 16) were the within-subject factors. In the extinction phase, the last 16 trials of three intensified groups and all 16 trials of the control group were analyzed, with *Stimulus* (CS+, CS-) and *Trial* (1 to 8) as the within-subject factors, to explore the efficacy of the different extinction protocols. Furthermore, the first 16 trials of the three intensified groups and 16 trials of the control group were analyzed in a control analysis, with *Stimulus* (CS+, CS-) as the within-subject factor, to exclude between-group extinction differences at the same stage of extinction learning. In the first and second recall phases, *Stimulus* (CS+, CS-) and *Trial* (1 to 8) served as the within-subject factors. In addition, the difference between SC responses to each CS+ and CS- across the four phases were compared by mixed model ANOVAs, with *Group* (4 groups) as the between-subject factor, and Trial (1 to 16 in acquisition, 1 to 8 in extinction and recall) as the within-subject factors. Also here, additionally the first 16 trials of three intensified groups and 16 trials of the control group in the extinction phase were analyzed by a one-way ANOVA as a control analysis, with *Group* (4 groups) as between-subject factor, and the SCR difference as the dependent variable.

As additional control analyses, a mixed model ANOVA was conducted including the three intensified groups to assess the comparative effects of spaced/massed extinction for each phase separately, with Stimulus (CS+, CS-) and Trial (1 to 16 in acquisition, 1 to 8 in extinction and recall) as the within-subject factors. The difference between SCR to each CS+ and CS- in the three intensified groups were also compared by mixed model ANOVAs, with Trial (1 to 16 in acquisition, 1 to 8 in extinction and recall) as the within-subject factors. All control analyses are presented in the supplementary material.

The rating of the last US and the likelihood of US occurrence were analyzed using a one-way ANOVA, with *Group* (4 groups) as between-subject factors, and rating scores as the dependent variable. Three distinct mixed model ANOVAs were conducted for the task-related questionnaires (valence, arousal, fear), with *Group* (4 groups) as the between-subject factor, and *Phase* (after acquisition, after extinction, after first recall, after second recall) and *Stimulus* (CS+, CS-) as within-subject factors.

## Results

### Demographics

The age, gender and education information of the participants and the applied US intensities did not show significant differences between groups. The detailed values are listed in Table 1.

**Psychological assessments:** The respective one-way ANOVAs revealed no significant differences between groups in the DASS-21 and ASI-3 (Table 1), suggesting similar psychological states of participants in the different groups.

**Rating of the US:** The one-way ANOVA comparing the last US rating in the fear acquisition phase showed no significant difference between groups (*p* = 0.313). In addition, there was no significant difference between participants in each group with respect to the perceived likelihood of US occurrence after CS+ and CS- presentation (0 % probability for CS- in 115 out of 117 participants) (Table 1).

**PANAS:** Three participants in the 3-hours spaced group did not provide data due to privacy concerns, thus the data of 114 participants were analyzed. The mixed model ANOVA revealed significant main effects of Phase (F (3, 330) = 18.873, *p* < 0.001) and Type (F (1, 110) = 111.573, *p* < 0.001). In addition, the interaction of Phase × Type was significant (F (2.7, 295.769) = 6.698, *p* < 0.001). There were no other significant main effects or interactions between groups (Table 2 and Table S4). Post-hoc tests showed that positive affect scores were significantly higher in the fear acquisition phase compared to the following extinction, and both extinction recall phases, while scores of the negative affect subscale did not show any significant changes across phases.

**Table 2:** Results of the mixed model ANOVA conducted for the positive and negative affect scale.

Factors	d.f., Error	*F value*	*η_p_^2^*	*p value*
Phase	3, 330	18.873	0.146	< 0.001*
Type	1, 110	111.573	0.504	< 0.001*
Group	3, 110	1.225	0.032	0.304
Phase × Group	9, 330	0.905	0.024	0.521
Type × Group	3, 110	0.255	0.007	0.858
Phase × Type	2.7, 295.769	6.698	0.057	< 0.001*
Phase × Type × Group	8.1, 295.769	1.234	0.033	0.278

⁎ Significant results at *p* < 0.05.

### Skin conductance response

#### SCR for each stimulus (CS+/CS-)

**Fig. 2:**
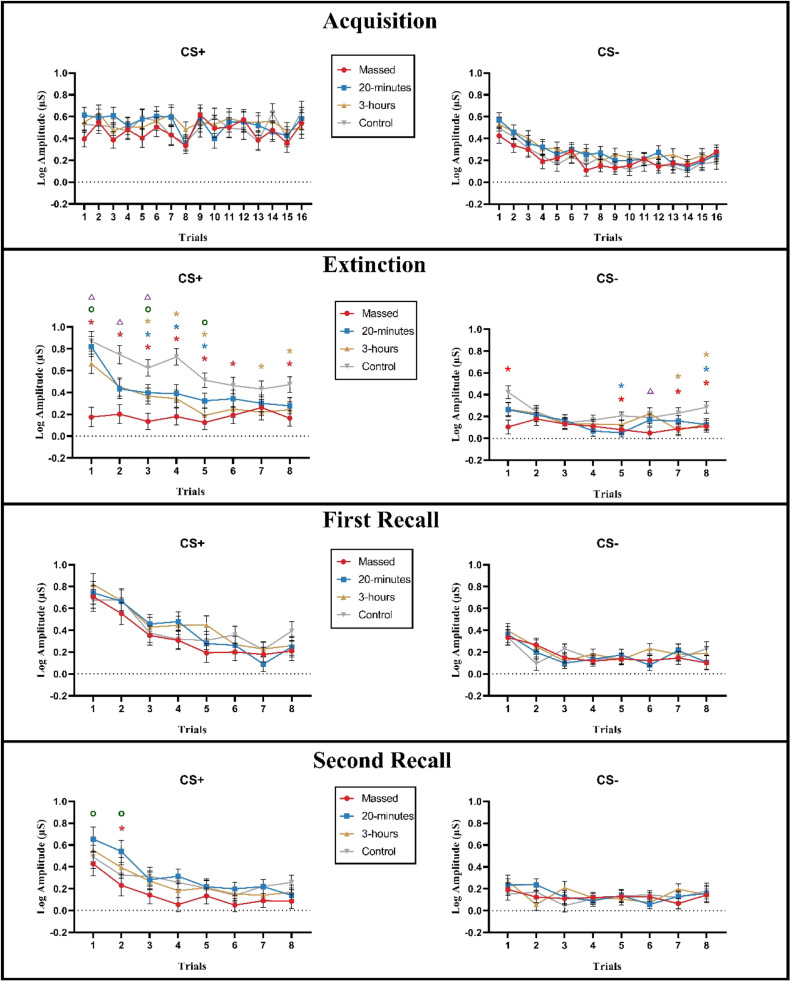
The SCR in response to the CS+ and CS- for each trial in acquisition, extinction, first and second recall phases are shown. The line color indicates the different extinction protocols, and error bars refer to the standard error of mean. In general, during the extinction phase, compared to the 16 trials of the control group, the last 16 trials of the massed and/or both spaced groups exhibited lower SCRs in response to the CS+ in the 1st to the 8th trials, and in response to the CS- in the 1st, 5th, 7th and 8th trials. Furthermore, the massed training showed lower SCRs in response to the CS+ (1st, 2nd, 3rd and 5th trials) and CS- (6th trial) compared to the 20-minutes and/or 3-hours spaced trainings. During the second recall phase, the massed training showed significantly lower SCRs in response to the CS+ in the 1st and 2nd trials compared to the 20-minutes spaced training, and in the 2nd trial compared to the control group. The symbols (*) indicate significant differences between the temporally distributed training conditions (red: massed, blue: 20-minutes, brown: 3-hours) and the control condition. The symbols (o) indicate significant differences between massed and 20-minutes spaced trainings, the symbols (△) indicate significant differences between massed and 3-hours spaced trainings, *p* < 0.05.

**Fear acquisition phase (Day 1):** The mixed model ANOVA revealed a significant main effect of Stimulus (F (1, 113) = 169.738, *p* < 0.001). The CS+ elicited significantly larger SCRs compared to the CS- (*p* < 0.001). In addition, the main effect of Trial was significant (F (10.7, 1210.486) = 8.760, *p* < 0.001). The interaction of Stimulus × Trial also showed a significant effect (F (11, 1245.727) = 7.592, *p* < 0.001) (Table 3 and Table S5). Post-hoc tests revealed that SCRs in response to the CS+ were larger compared to those in response to the CS- from the 2nd to the 16th trial (*p* < 0.05). These results indicate that participants quickly recognized the CS+ as the threat cue and the CS- as the safety cue during acquisition learning (Fig. 2).

**Table 3:** Results of the mixed model ANOVAs for comparing SCR in acquisition, extinction (the last 16 trials of the three intensified groups and 16 trials of the control group), first and second recall phases.

	Factors	d.f., Error	*F value*	*η_p_^2^*	*p value*
Acquisition	Group	3, 113	0.533	0.014	0.660
	Stimulus	1, 113	169.738	0.600	< 0.001*
	Trial	10.7, 1210.486	8.760	0.072	< 0.001*
	Stimulus × Group	3, 113	0.241	0.006	0.867
	Trial × Group	32.1, 1210.486	0.786	0.020	0.799
	Stimulus × Trial	11, 1245.727	7.592	0.063	< 0.001*
	Stimulus × Trial × Group	33.1, 1245.727	1.049	0.027	0.393
Extinction	Group	3, 113	6.976	0.156	< 0.001*
	Stimulus	1, 113	86.173	0.433	< 0.001*
	Trial	5.1, 577.098	24.062	0.176	< 0.001*
	Stimulus × Group	3, 113	7.145	0.159	< 0.001*
	Trial × Group	15.3, 577.098	2.963	0.073	< 0.001*
	Stimulus × Trial	5.9, 669.362	6.131	0.051	< 0.001*
	Stimulus × Trial × Group	17.8, 669.362	2.358	0.059	< 0.001*
First recall	Group	3, 113	0.398	0.010	0.755
	Stimulus	1, 113	82.296	0.421	< 0.001*
	Trial	5, 564.418	40.606	0.264	< 0.001*
	Stimulus × Group	3, 113	0.522	0.014	0.668
	Trial × Group	15, 564.418	0.817	0.021	0.659
	Stimulus × Trial	5.3, 601.304	14.671	0.115	< 0.001*
	Stimulus × Trial × Group	16, 601.304	1.384	0.035	0.144
Second recall	Group	3, 113	0.792	0.021	0.501
	Stimulus	1, 113	35.122	0.237	< 0.001*
	Trial	5.2, 590.460	22.236	0.164	< 0.001*
	Stimulus × Group	3, 113	2.808	0.069	< 0.043*
	Trial × Group	15.7, 590.460	1.138	0.029	0.316
	Stimulus × Trial	5.6, 627.722	8.646	0.071	< 0.001*
	Stimulus × Trial × Group	16.7, 627.722	0.859	0.022	0.621

⁎ Significant results at *p* < 0.05.

**Extinction learning phase (Day 2):** The mixed model ANOVA conducted over the last 16 trials of the three intensified groups and all 16 trials of the control group revealed a significant main effect of Group (F (3, 113) = 6.976, *p* < 0.001). Post-hoc test showed that the massed and both spaced groups had significantly lower SCRs compared to the control group. Furthermore, SCRs were significantly smaller in the massed group compared to the 20-minutes spaced group (*p* = 0.028). In addition, the main effects of Stimulus (F (1, 113) = 86.173, *p* < 0.001) and Trial (F (5.1, 577.098) = 24.062, *p* < 0.001) were significant. The Stimulus × Group interaction was also significant (F (3, 113) = 7.145, *p* < 0.001). The post-hoc tests showed that SCRs in response to the CS+ were significantly smaller in the massed and both spaced groups compared to the control group. Furthermore, the massed group showed significantly smaller SCRs in response to the CS+ compared to the 20-minutes spaced group, and significantly smaller SCRs in response to the CS- compared to the control group. Furthermore, the Trial × Group (F (15.3, 577.098) = 2.963, *p* < 0.001) and Stimulus × Trial (F (5.9, 669.362) = 6.131, *p* < 0.001) interactions were significant. In addition, the Stimulus × Trial × Group interaction showed a significant effect (F (17.8, 669.362) = 2.358, *p* < 0.001) (Table 3, Table S6 and Fig. 2). The post-hoc tests revealed that, in comparison to the control condition, 1) the massed group showed significantly smaller SCRs in response to the CS+ from the 1st to the 8th trial, except for the 7th trial, and in response to the CS- in the 1st, 5th, 7th and 8th trials (*p* < 0.05); 2) the spaced training group with an interval of 20 minutes showed significantly smaller SCRs in response to the CS+ from the 3rd to the 5th trial, and in response to the CS- in the 5th and 8th trial; 3) the 3-hours spaced group showed significantly smaller SCRs in response to the CS+ from the 3rd to 5th, and from 7th to the 8th trial, and in response to the CS- in the 7th and 8th trials. Further analyses revealed a significant reduction of SCRs in response to the CS+ in the massed group in the 1st, 3rd, and 5th trials in comparison to the 20 min spaced group. Relative to the 3-hours spaced group, the massed group showed significantly reduced SCRs in response to the CS+ from the 1st to 3rd trials, and in response to the CS- in the 6th trial (*p* < 0.05). These results indicate that both massed and spaced trainings enhanced extinction learning compared to the control group. Furthermore, the massed group was more efficient than both spaced groups.

**First recall phase (Day 3):** The mixed model ANOVA showed significant main effects of Stimulus (F (1, 113) = 82.296, *p* < 0.001), Trial (F (5, 564.418) = 40.606, *p* < 0.001), and the Stimulus × Trial interaction (F (5.3, 601.304) = 14.671, *p* < 0.001) (Table 2). No other significant main effects or interactions were observed (Table 3, Table S6 and Fig. 2). Post-hoc tests indicated that except for the 7th trial, the CS+ revealed significantly larger SCRs compared to the CS- (*p* < 0.05), and the SCR to CS+ and CS- in the 1st trial were significantly larger compared to all other trials (*p* < 0.05).

**Fig. 3:**
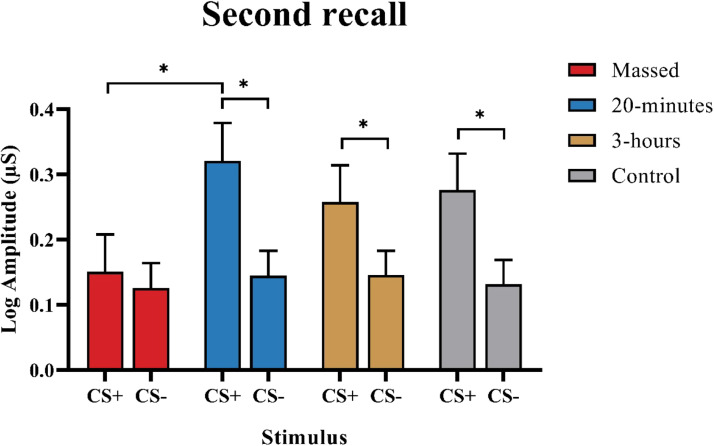
SCR values averaged over all trials of the second recall phase, shown separately for CS+ and CS-. The color of the bars represents different extinction training paradigms, with error bars indicating the standard error of the mean. In general, the mean SCR in response to the CS+ was significantly lower in the massed group compared to the 20-minutes spaced training (*p* = 0.04). Only the massed group showed no significant difference between SCRs in response to the CS+ and CS- (*p* = 0.524). The * shows significant results of the post hoc LSD tests (*p* < 0.05).

**Second recall phase (Day 4):** The mixed model ANOVA revealed significant main effects of Stimulus (F (1, 113) = 35.122, *p* < 0.001), Trial (F (5.2, 590.460) = 22.236, *p* < 0.001), and significant Stimulus × Trial (F (5.6, 627.722) = 8.646, *p* < 0.001), and Stimulus × Group (F (3, 113) = 2.808, *p* = 0.043) interactions (Table 3, Table S6 and Fig. 2). Post-hoc tests showed that SCRs in response to the CS+ were significantly smaller in the massed compared to the 20-minutes spaced group (*p* = 0.04). Furthermore, only the massed group showed no significant SCR difference in response to the CS+ and CS- (*p* = 0.524) (Fig. 3). These results indicate that only in the massed group extinction remained stable after one week.

#### The SCR difference between CS+ and CS-

**Fig. 4:**
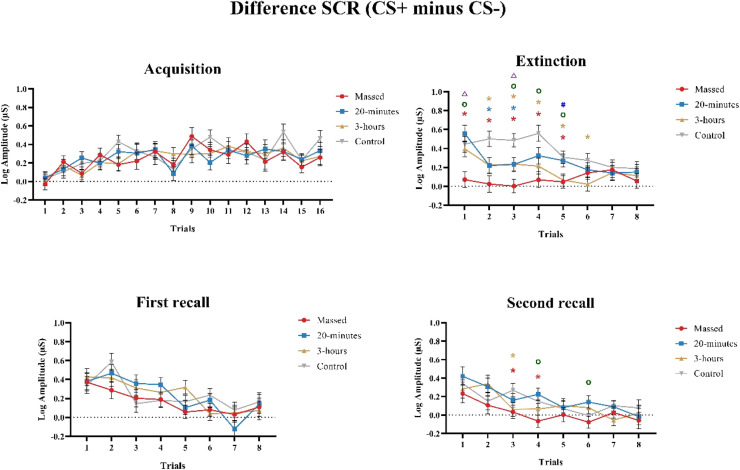
The difference between SCR in response to each CS+ and CS- presentation across the four phases of the study. The lines represent different training conditions during extinction, and error bars refer to the standard error of the mean. In general, during the extinction phase, compared to the control group, the massed and/or both spaced groups showed a lower SCR difference in the 1st to the 6th trials. Furthermore, the massed group showed a lower SCR difference in the 1st, 3rd, 4th and 5th trials compared to the 20-minutes and/or 3-hours spaced conditions. The 3-hours spaced group showed a lower SCR difference in the 5th trial compared to the 20-minutes spaced group. During the second recall phase, the massed and/or 3-hours spaced groups showed a significant lower SCR difference in the 3rd and 4th trials compared to the control group. The massed group also showed a lower SCR difference in the 4th and 6th trials than the 20-minutes spaced training. The symbols (*) indicate significant differences between the temporally distributed training conditions (red: massed, blue: 20-minutes, brown: 3-hours) and the control condition. The symbols (o) indicate significant differences between massed and 20-minutes spaced trainings, the symbols (△) indicate significant differences between massed and 3-hours spaced trainings, the symbols (#) indicate significant differences between 20-minutes and 3-hours spaced trainings, *p* < 0.05.

In a further analysis, mixed model ANOVAs were conducted to investigate difference values between SCR in response to each CS+ and CS- across the four phases. The results are shown in Table 4, Table S7 and Fig. 4.

**Table 4:** Results of the mixed model ANOVAs conducted for the difference between SCR generated by each CS+ and CS- presentation across acquisition, extinction, first and second recall phases.

	Factors	d.f., Error	F value	η_p_^2^	p value
Acquisition	Trial	11, 1245.727	7.592	0.063	< 0.001*
	Group	3, 113	0.241	0.006	0.867
	Trial × Group	33.1, 1245.727	1.049	0.027	0.393
Extinction	Trial	5.9, 669.362	6.131	0.051	< 0.001*
	Group	3, 113	7.145	0.159	< 0.001*
	Trial × Group	17.8, 669.362	2.358	0.059	0.001*
First recall	Trial	5.3, 601.304	14.671	0.115	< 0.001*
	Group	3, 113	0.522	0.014	0.668
	Trial × Group	16, 601.304	1.384	0.035	0.144
Second recall	Trial	5.6, 627.722	8.646	0.071	< 0.001*
	Group	3, 113	2.808	0.069	0.043*
	Trial × Group	16.7, 627.722	0.859	0.022	0.621

Significant results are marked with asterisks.

**Acquisition:** The results revealed a significant main effect of Trial (F (6.1, 689.352) = 8.547, *p* < 0.001). No other significant main effects or interactions were revealed.

**Extinction:** The results showed significant main effects of Trial (F (5.9, 669.362) = 6.131, *p* < 0.001) and Group (F (3, 113) = 7.145, *p* < 0.001), and a significant Trial × Group interaction (F (17.8, 669.362) = 2.36, *p* = 0.001). The post-hoc tests indicated that, in comparison to the control condition, 1) the massed group showed significantly smaller SCRs from the 1st to the 5th trial (*p* < 0.05); 2) the 20-minutes spaced group showed significantly smaller SCRs in the 2nd and 3rd trials (*p* = 0.02 and *p* = 0.015, respectively); 3) the 3-hours spaced group showed significantly smaller SCRs from the 2nd to the 6th trial (*p* < 0.05). Further analyses showed a significantly larger reduction of SCRs in the massed group in the 1st, 3rd, 4th and 5th trials than the 20-minutes spaced group. Relative to the 3-hours spaced group, the massed group showed significantly lower SCRs in the 1st and 3rd trials (*p* = 0.07 and *p* = 0.024, respectively). In addition, the 3-hours spaced group showed a significantly lower SCR in the 5th trial compared to the 20-minutes spaced group (*p* = 0.028). These results reconfirm that both massed and spaced training enhanced extinction learning compared to the control group. Moreover, massed training showed superior extinction performance compared to both spaced training protocols.

**First recall:** The results revealed a significant main effect of Trial (F (5.3, 601.304) = 14.671, *p* < 0.001). No other significant main effects or interactions were revealed (Table 4).

**Second recall:** The results revealed significant main effects of Trial (F (5.6, 627.722) = 8.646, *p* < 0.001), and Group (F (3, 113) = 2.808, *p* = 0.043). Post-hoc test indicated that massed training showed significantly lower SCR compared to the 20-minutes spaced and control groups (*p* = 0.007 and *p* = 0.03, respectively). This result suggests that the massed training effects persisted until at least one week after extinction learning.

### Questionnaires

**Fig. 5:**
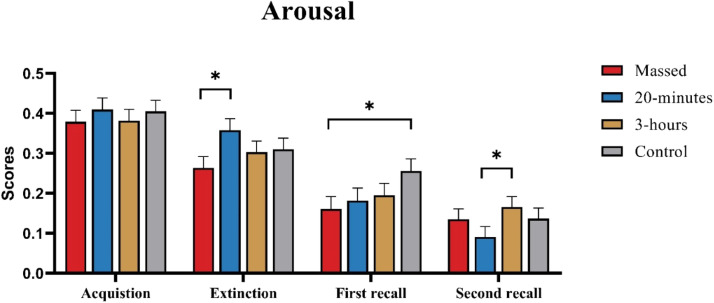
Arousal rating scores in each study phase for all four groups, combined for CS+ and CS-. The colors of the bars represent different extinction training conditions, and error bars depict the standard error of the mean. In general, the massed training resulted in a lower arousal rating compared to the 20-minutes spaced group during the extinction phase, and in the first recall, the massed training showed lower ratings compared to the control group (*p* < 0.05). In addition, the 20-minutes spaced group showed a lower arousal rating compared to the 3-hours spaced group during the second recall phase (*p* < 0.05). The asterisks show significant results (*p* < 0.05).

**Valence, arousal, and fear ratings of the CS+ and CS-:** Three mixed model ANOVAs comparing valence, arousal, and fear ratings of the CS+ and CS- across the different phases (acquisition, extinction, first and second recall) of the study showed significant main effects of Phase, Stimulus, and a significant Stimulus × Phase interaction. The post-hoc tests revealed significant differences between stimuli in each phase (*p* < 0.001), indicating that the participants associated the CS+ with reduced pleasantness, increased arousal, and elevated levels of fear compared to the CS- across all phases. In addition, the Group × Phase interaction was significant with respect to arousal ratings (F (6.7, 252.045) = 2.113, *p* = 0.045). Post-hoc tests showed that the massed group reported a lower level of arousal compared to the 20-minutes spaced group after extinction (*p* = 0.021) and compared to the control group after the first recall phase (*p* = 0.029). After the second recall phase, the 20-minutes spaced group showed significantly lower arousal ratings compared to the 3-hours spaced group (*p* = 0.044) (Fig. 5). More details are shown in Table 5, Table S8 and Fig S2.

**Table 5:** Results of the mixed model ANOVAs for valence, arousal and fear ratings.

	Factors	d.f., Error	F value	η_p_^2^	p value
Valence	Stimulus	1, 113	509.942	0.819	< 0.001*
	Phase	2.7, 307.527	78.352	0.409	< 0.001*
	Group	3, 113	0.210	0.006	0.889
	Stimulus × Group	3, 113	0.598	0.016	0.618
	Phase × Group	8.2, 307.527	1.824	0.046	0.070
	Stimulus × Phase	2.4, 273.521	62.170	0.355	< 0.001*
	Stimulus × Phase × Group	7.3, 273.521	1.330	0.034	0.234
Arousal	Stimulus	1, 113	425.393	0.790	< 0.001*
	Phase	2.2, 252.045	109.368	0.492	< 0.001*
	Group	3, 113	0.703	0.018	0.552
	Stimulus × Group	3, 113	0.223	0.006	0.880
	Phase × Group	6.7, 252.045	2.113	0.053	0.045*
	Stimulus × Phase	2.2, 243.016	46.373	0.291	< 0.001*
	Stimulus × Phase × Group	6.5, 243.016	1.201	0.031	0.305
Fear	Stimulus	1, 113	369.080	0.766	< 0.001*
	Phase	2.4, 265.805	103.783	0.479	< 0.001*
	Group	3, 113	1.435	0.037	0.236
	Stimulus × Group	3, 113	1.077	0.028	0.694
	Phase × Group	7.1, 265.805	1.704	0.043	0.107
	Stimulus × Phase	2.2, 242.769	60.179	0.347	< 0.001*
	Stimulus × Phase × Group	6.5, 242.769	1.567	0.040	0.152

⁎ Significant results at *p* < 0.05.

## Discussion

In this study, we investigated the impact of massed training and two different intervals of spaced training on fear extinction learning and extinction retention in a sample of healthy participants. Physiological response (SCR) and subjective experiences were recorded in a Pavlovian fear conditioning paradigm.

Our main findings reveal that, for SCR, the massed and both spaced training improved the efficiency of extinction learning compared to classical extinction training, and the massed group demonstrated superior extinction performance relative to both spaced groups in the extinction phase. In the second recall phase, only the massed group did not show fear responses to the threat cues, implying that the massed group retained extinction memory for at least one week. Furthermore, with respect to arousal ratings in the questionnaire data, participants in the massed extinction group reported lower arousal compared to the classical extinction training after the first recall phase. These observations indicate that both massed and spaced training facilitated extinction learning, with only massed training showing both short- and long-term extinction retention.

The main result of the present study, namely that massed extinction training resulted in superior extinction learning and retention compared to spaced training, is not in accordance with our initial hypotheses. This might be caused by the complex feature of the extinction process. Generally, spaced training is beneficial in various types of learning (Carpenter et al., 2012; Donovan & Radosevich, 1999; Shea et al., 2000; Smolen et al., 2016). However, fear extinction is more complex than the typical learning process, because it involves not only the formation of new memories but also the retrieval of specific past memories (Lovibond, 2004). Previous research has shown that the CS presentation triggers two opposing processes during extinction learning: the emergence of extinction and the retrieval of the original fear memory. It was shown that massed CS presentations primarily elicit the extinction process, whereas spaced CS presentations trigger both extinction process and fear memory retrieval (Cain, Blouin, & Barad, 2004). In terms of the mechanism of LTP for fear extinction, increased LTP in the amygdala and hippocampus strengthens the formation and long-term retention of fear memory. Conversely, the enhancement of LTP in the mPFC suppresses fear memory and facilitates long-term extinction memory (Kim & Jung, 2006; Luchkina & Bolshakov, 2019). Spaced training has been shown to enhance LTP in the hippocampus, a process dependent on protein synthesis, which strengthens long-term fear memory. However, massed training did not enhance hippocampal LTP (Scharf et al., 2002). In addition, a previous study showed that LTP in the mPFC was enhanced during the latter part of massed extinction training and lasted for at least 24 h after extinction (Herry et al., 1999). However, in another study of the same team, LTP in the mPFC was enhanced during the last of three spaced extinction sessions but returned to baseline values after 24 h (Herry & Garcia, 2002). This suggests that spaced extinction training might trigger both, the consolidation of fear memory and new extinction learning. In contrast, massed training appears to primarily facilitate extinction learning, resulting in long-term extinction memory.

The underlying mechanisms of the effect of different temporal distributions on extinction learning remains however partially unclear, as current studies often show heterogeneous results (Jiang et al., 2016; Tapias-Espinosa et al., 2018). While our findings suggest that massed training enhances long-term extinction retention, previous animal research has shown partially different outcomes. Specifically, an animal study showed that spaced extinction training with a 7-day interval reduced fear recovery 28 days after extinction, compared to massed training with a 24-hour interval. The authors found that spaced training increased activation in the infralimbic cortex, which is known to be critical for the consolidation of extinction (Moody, Sunsay, & Bouton, 2006; Tapias-Espinosa et al., 2018). Conversely, another rat study showed that massed extinction training, compared to 30-minutes interval spaced training, increased Rac1 activity, a protein that plays a key role in shaping neurons and supporting memory formation (Dietz et al., 2012), in the hippocampus one hour after training. This increase in Rac1 activity induced behavioral long-term extinction (for at least 16 days) of contextual fear in rats. However, spaced training was found to suppress Rac1 activation and impair extinction (Jiang et al., 2016). In a further study of the same team, spaced extinction training was shown to enhance context-related fear memory by increasing the expression of hippocampal 5-HT2A receptors, whose activation suppresses Rac1 activity (Jiang et al., 2019). These seemingly conflicting findings suggest that the effects of the temporal distributions on fear extinction might depend on several factors, including fear conditioning protocols, individual differences, and experimental design. Since our study primarily focused on behavioral findings, future research should incorporate neuroimaging methods to better elucidate the underlying neural mechanisms, and explore different exposure intervals in a systematic way.

In addition, it is suggested that extinction only occurs when experiences contradict existing contingency expectations—if the expectation that the CS leads to the US is firm and protected, extinction might not happen (Hofmann, 2008). This means that extinction learning involves inhibiting the original fear-related CS-US association and forming a new CS-no US association (Bouton, 1993). It has been shown that repeated CS presentations without the US during extinction learning leads to extinction of the fear response (Rescorla & Heth, 1975). This is possibly caused by the strengthening of the CS-no US association. The outcome of massed training in the present study supports the view that repeated and sustained multiple exposures enhance extinction learning and consolidation. However, the finding of the present study that spaced training elicited a relevant fear response to the CS+ during the second extinction session may imply a recovery of the original fear-inducing CS-US association. It has been shown that substantial exposure is necessary for extinction formation (Hepp, Pérez-Cuesta, Maldonado, & Pedreira, 2010). Although the total number of trials was the same for both massed and spaced training in this study, the interval between extinction sessions might cause the new CS-no US association to compete with the recovered original CS-US association. This competition might interrupt the stabilization of the CS-no US association, resulting in reduced consolidation of extinction memory.

Context has been recognized as a factor impacting the effectiveness of spaced extinction learning (Miguez, Witnauer, Laborda, & Miller, 2014; Urcelay, Wheeler, & Miller, 2009). When extinction occurs in the acquisition context, which is strongly associated with the US, spaced training resulted in more fear responses compared to massed training. In contrast, in a novel context that lacks association with the US, spaced training led to less fear responses than massed training (Miguez et al., 2014; Morris, Furlong, & Westbrook, 2005). It is suggested that the response to the CS is associated with two sources: it is directly related to the strength of the original CS-US association and indirectly related to the context-US association. Spaced training might consequently result in a learning benefit in facilitating extinction learning in a context with low associative value due to weak or no context-US association. Conversely, in an extinction context with high associative value, the benefit of spaced training is contrasted by the strong context-US association (Miguez et al., 2014). Therefore, since the extinction context was identical to the acquisition context in the present study, this high correlation value resulted in a strong context-US linkage, which might weaken the extinction effectiveness of spaced training.

The results of the three intensified group comparisons suggest that the timing of extinction training has an important effect on extinction learning and retention. Our results also show that both massed and spaced training facilitate extinction learning more than a conventional control protocol. This finding may be related to the number of exposures. An animal study revealed that tripling the number of trials during extinction enhanced the effectiveness of fear extinction compared to tripling the duration of each trial. The authors suggested that extinction is more event-driven than time-based, with conditioned responses decreasing as the number of extinction trials increases. It was suggested that each trial during extinction is perceived as a single episode, and every time a trial without threat happens, the animal re-evaluates the response towards the CS (Chan & Harris, 2017). In accordance, when the total exposure duration during extinction was identical, a larger number of short-duration trials led to faster extinction compared to a lower number of trials with longer durations (Golkar, Bellander, & Öhman, 2013; Harris & Andrew, 2017; Haselgrove & Pearce, 2003). Based on these assumptions, it can be inferred that both the massed and spaced protocols improved performance versus the conventional protocol because of a larger number of exposures during extinction, thus resulting in enhanced extinction learning.

Furthermore, the results of the post-task self-report questionnaires showed that participants in the massed training group showed lower arousal levels during the first recall phase compared to those in the conventional training group. This finding suggests that massed extinction training, through high-frequency, continuous exposure, is more effective in reducing subjective fear in the short term. However, at the second recall phase one week later, no significant differences between the temporally distributed training conditions and the control group were observed. In contrast, the SCR results demonstrated that massed training results in a lasting extinction retention at the physiological level, showing its long-term inhibitory effect on autonomic nervous system activity. Presumably, this discrepancy might be caused by the fact that the questionnaires are primarily determined by subjective perceptions (Wallbott & Scherer, 1989), which may normalize or diminish after one week. It has been shown that implicit memory tends to last longer than explicit memory (Bailey et al., 2015). Self-report questionnaires are considered to be closely related to explicit memory, reflecting conscious recall and evaluation of the conditioned stimuli, whereas SCR is strongly related to implicit memory, indicating unconscious emotional and physiological responses (Madaboosi, Grasser, Chowdury, & Javanbakht, 2021; Packard, Rodríguez-Fornells, Stein, Nicolás, & Fuentemilla, 2014).

Our findings, however, contrast with some previous clinical studies that have shown superior learning and memory benefits with spaced training (Rowe & Craske, 1998; Tsao & Craske, 2000). One potential explanation for this difference is the saliency of the stimulus. Studies suggest that salient stimuli play a crucial role in learning and memory formation by impacting brain activity, attention, and cognitive performance (Santangelo & Macaluso, 2013; Taylor, Phan, Decker, & Liberzon, 2003; Zink, Pagnoni, Chappelow, Martin-Skurski, & Berns, 2006). In the present study, the salience of each CS presentation in the massed training condition might be enhanced by its sustained and dense exposure. This high salience stimuli should have facilitated rapid and effective adaptation and habituation to the threat cues, thus significantly inhibiting fear memory. In contrast, intervals in spaced training might diminish stimulus saliency, impairing the suppression of fear memory. However, in clinical studies, each stimulus might achieve higher salience through more realistic presentation, larger emotional engagement, and stronger relevance (Magliacano, De Bellis, Galvao-Carmona, Estraneo, & Trojano, 2019; Trojano, Moretta, Loreto, Santoro, & Estraneo, 2013). This variation in stimulus saliency may be a critical factor in the distinct effects of massed and spaced training. Future studies could investigate the role of stimulus saliency in different extinction training protocols and its effects on fear extinction learning and retention. In addition, individual differences often cause divergence of results (Battig, 1979). The participants in the present study were healthy, young adults with stable mental states and no psychological disorders, which might account for the differences observed between our laboratory results and clinical studies. In future studies, recruiting and comparing patients with anxiety or fear-related symptoms to further evaluate intervention efficacy in clinical samples might be relevant. Furthermore, the intervals used for spaced conditions in this study were typically shorter than those employed in previous clinical studies (Foa et al., 2018; Rowe & Craske, 1998). The duration of intervals has been shown to influence the long-term memory retention of spaced training (Cepeda et al., 2006; Tapias-Espinosa et al., 2018). For example, spaced training with intervals that include sleep may involve positive effects of sleep on memory consolidation (Bell, Kawadri, Simone, & Wiseheart, 2014). In summary, clinical studies often involve many complex variables, such as diverse patient populations, varying degrees of symptom severity, and the complexity of real-world situations. These factors might significantly impact the efficacy of spaced protocols observed in clinical settings. In contrast, physiological studies are conducted under highly controlled conditions with few well controlled factors. This discrepancy might account for the challenge in directly translating physiological findings into clinical research. Thus, a systematic evaluation of specific extinction protocols is essential to bridge the gap between laboratory findings and clinical applications.

### Limitations and future directions

Some limitations of the present study should be taken into account. First, we recorded only SCR data and questionnaires as primary outcome measurements. Integrating additional measures, such as fear-potentiated startle and pupillary responses, would allow for a more comprehensive understanding of the physiological process involved in fear extinction (Lonsdorf et al., 2017). These measures provide further insights into the autonomic nervous system response and might enhance our understanding of the emotional and attentional aspects of the extinction process. Second, in this study, the follow-up test was conducted once after one week. To gain a more detailed overview of the dynamics and time course of the effects of massed and spaced training, it would be beneficial to conduct follow-up tests at shorter intervals, such as 3 days after extinction, as well as at longer intervals similar to clinical studies (Rowe & Craske, 1998; Tsao & Craske, 2000), such as 1 month after intervention. Furthermore, the control group in this study was exposed to a relatively low number of extinction trials compared to the experimental group. Incorporating a higher number of trials in the control group during the extinction phase might provide a more nuanced understanding of the effects of different training protocols on extinction learning and retention (Bach et al., 2023). Last but not least, utilizing neuroimaging techniques to observe the activation and functional connectivity of cerebral areas during massed and spaced training would yield deeper insights into the neural mechanisms underlying specific fear extinction protocols, and diverging outcomes.

In this study, although spaced extinction training did not show a superior effect compared to massed training, its potential to facilitate long-term memory remains worthy of further investigation. Evidence indicates that a higher number of extinction trials, resulting in longer total exposure, improves the retention of extinction memory (Foa et al., 2005; Foa & Rauch, 2004; Hepp et al., 2010; Maples-Keller et al., 2022). Prolonged exposure (PE) therapy, which employs fear extinction as one of its primary mechanisms, has been shown to facilitate corrective learning (Association, 2017). Repeatedly activating negative emotions within a safe context might allow patients to reassess and integrate information that contradicts expected harm. This process is efficient to alter the negative cognitions related to trauma in PTSD patients (Cooper, Clifton, & Feeny, 2017; McLean & Foa, 2022). Studies have found that PTSD patients receiving PE therapy showed a decrease in negative thoughts for up to 12 months after intervention (Foa et al., 2005; Foa & Rauch, 2004). Thus, increasing the number of trials in each single session of spaced training may help to consolidate the newly formed, fragile extinction memory, and reduce the likelihood of disturbance from the original fear memory. Moreover, adding additional trials to massed training may also positively impact long-term memory consolidation. Exploring and comparing the effects of a further enhanced number of trials for both massed and spaced training might be a worthwhile direction for future research.

## Conclusion

The results of the current study suggest that both massed and spaced training promote extinction learning compared with a conventional protocol, with massed training yielding stronger effects. Furthermore, only massed training resulted in lower arousal ratings in the first recall and maintained extinction for at least one week after intervention. These findings suggest that extinction training with extensive and continuous stimulus presentation enhances the efficacy of extinction learning and stabilizes extinction. In contrast, intervals during the extinction learning process may also trigger the consolidation of fear memory, negatively impacting extinction retention. Moreover, since changes in the extinction context may alter the efficacy of massed and spaced training, a systematic evaluation of specific extinction protocols would be relevant. Future studies should explore the mechanisms of these effects by integration of neuroimaging techniques to investigate the neural connectivity and activation patterns in the brain associated with specific extinction protocols, and explore the transferability of these results to respective patients, and clinical applications.

## CRediT authorship contribution statement

**Yuanbo Ma:** Conceptualization, Investigation, Data curation, Formal analysis, Visualization, Writing – original draft, Writing – review & editing. **Dzheylyan Kyuchukova:** Investigation, Data curation. **Fujia Jiao:** Data curation, Visualization, Writing – review & editing. **Giorgi Batsikadze:** Methodology. **Michael A. Nitsche:** Conceptualization, Funding acquisition, Project administration, Supervision, Methodology, Writing – review & editing. **Fatemeh Yavari:** Conceptualization, Methodology, Resources, Software, Project administration, Supervision, Validation, Writing – review & editing.

## Declaration of competing interest

The authors declare the following financial interests/personal relationships which may be considered as potential competing interests:

Michael A Nitsche reports a relationship with Neuroelectrics that includes: consulting or advisory. Michael A Nitsche reports a relationship with Precisis that includes: consulting or advisory. If there are other authors, they declare that they have no known competing financial interests or personal relationships that could have appeared to influence the work reported in this paper.

## Appendix Supplementary materials

Image, application 1
