# Supplementary material for: The impact of temporal distribution on fear extinction learning
Source: Int J Clin Health Psychol. 2025 Jan 11;25(1):100536. doi: 10.1016/j.ijchp.2024.100536 (PMC11770545; doi:10.1016/j.ijchp.2024.100536)

**Supplementary materials**

**Results of the control analysis**

***Skin conductance response***

**SCR for each stimulus (CS+/CS-)**

**Extinction learning phase:** As a control analysis, in the first 16 trials of the three intensified groups and 16 trials of the control group, the mixed model ANOVA showed a significant main effect of Stimulus (F (1, 113) = 213.935, p < 0.001). No other significant main effects or interactions were observed (Table S1 and Figure S1).

**The SCR difference between CS+ and CS-**

**Extinction:** In the respective control analysis, in the first 16 trials of the three intensified groups and 16 trials the control group, the one-way ANOVA showed no significant effect of extinction protocol on the SCR difference at the p < 0.05 level for the four conditions (F (3, 116) = 0.346, p = 0.792).

**SCR for each stimulus (CS+/CS-) in the three intensified groups**

**Fear acquisition:** The mixed model ANOVA revealed significant main effects of Stimulus (F (1, 84) = 109.625, p < 0.001) and Trial (F (9.9, 835.149) = 6.524, p < 0.001). The interaction of Stimulus × Trial also showed a significant effect (F (10.3, 861.914) = 4.985, p < 0.001). Post-hoc tests revealed that SCRs in response to the CS+ were larger compared to those in response to the CS- from the 2^nd^ to the 16^th^ trial (p < 0.05). No other significant main effects or interactions were observed (Table S2).

**Extinction learning:** In the first 16 extinction trials of the three intensified groups, the mixed model ANOVA showed significant main effects of Stimulus (F (1, 84) = 156.969, p < 0.001) and Trial (F (5.1, 427.530) = 46.903, p < 0.001). The interaction of Stimulus × Trial also showed a significant effect (F (4.3, 359.916) = 15.320, p < 0.001). Post-hoc tests revealed that SCRs in response to the CS+ were larger compared to those in response to the CS- from the 1st to the 8th trial (p < 0.05). No other significant main effects or interactions were observed (Table S2).

In the second 16 extinction trials of the three intensified groups, the results showed significant main effects of Stimulus (F (1, 84) = 39.409, p < 0.001) and Trial (F (4.8, 399.444) = 16.988, p < 0.001). The Stimulus × Group (F (2, 84) = 3.905, p = 0.024), Trial × Group (F (9.5, 399.444) = 4.112, p < 0.001), Stimulus × Trial (F (5.5, 458.697) = 4.477, p < 0.001) and Trial × Stimulus × Group (F (10.9, 458.697) = 2.392, p = 0.007) interactions were also significant (Table S2). Post-hoc tests showed that SCRs in response to the CS+ were significantly smaller in the massed group from the 1st to the 3rd trial compared to the 3-hours spaced training, and in the 1st trial and from the 3rd to the 5th trial compared to the 20-minutes spaced training. In addition, the massed group showed a significantly smaller SCR in response to the CS- in the 6th trial compared to the 3-hours spaced training.

**First recall:** The mixed model ANOVA revealed significant main effects of Stimulus (F (1, 84) = 57.214, p < 0.001) and Trial (F (5.1, 426.346) = 34.994, p < 0.001). The interaction of Stimulus × Trial also showed a significant effect (F (5, 415.794) = 11.486, p < 0.001). Post-hoc tests indicated that except for the 7th and 8th trial, the CS+ revealed significantly larger SCR compared to the CS- (p < 0.05). No other significant main effects or interactions were observed (Table S2).

**Second recall:** Second recall phase (Day 4): The mixed model ANOVA revealed significant main effects of Stimulus (F (2, 84) = 26.432, p < 0.001), Trial (F (4.7, 396.116) = 19.893, p < 0.001), and significant Stimulus × Trial (F (4.5, 380.942) = 8.152, p < 0.001), and Stimulus × Group (F (2, 84) = 4.602, p = 0.013) interactions (Table S2). Post-hoc tests showed that SCRs in response to the CS+ were significantly smaller in the massed compared to the 20-minutes spaced group (p = 0.046). Furthermore, only the massed group showed no significant SCR difference in response to the CS+ and CS- (p = 0.483).

**The SCR difference between CS+ and CS- in the three intensified groups**

**Acquisition:** The mixed ANOVA revealed a significant main effect of Trial (F (10.3, 861.914) = 4.985, p < 0.001). No other significant main effects or interactions were revealed (Table S3).

**Extinction:** In the first 16 extinction trials of the three intensified groups, the mixed model ANOVA showed a significant main effect of Trial (F (4.3, 359.916) = 15.320, p < 0.001). No other significant main effects or interactions were revealed (Table S3).

In the second 16 extinction trials of the three intensified groups, the results showed significant main effects of Trial (F (5.5, 458.697) = 4.477, p < 0.001) and Group (F (2, 84) = 3.905, p = 0.024), and a significant Trial × Group interaction (F (10.9, 458.697) = 2.392, p = 0.007) (Table S3). The post-hoc tests showed a significantly larger reduction of SCR in the massed group in the 1st, 3rd, 4th and 5th trials than the 20-minutes spaced training, and in the 1st and 3rd trials compared to the 3-hours spaced training (p = 0.07 and p =0.016, respectively). In addition, the 3-hours spaced training showed a significantly lower SCR in the 5th trial compared to the 20-minutes spaced training (p = 0.017).

**First recall:** The results revealed a significant main effect of Trial (F (5, 415.794) = 11.486, p < 0.001). No other significant main effects or interactions were revealed (Table S3).

**Second recall:** The results revealed significant main effects of Trial (F (4.5, 380.942) = 8.152, p < 0.001), and Group (F (2, 84) = 4.602, p = 0.013). Post-hoc tests indicated that massed training showed significantly lower SCR compared to the 20-minutes spaced training (p = 0.003). No other significant main effects or interactions were revealed (Table S3).

| **Table S.1.** Results of the mixed model ANOVA conducted for the control analysis of comparing SCR in extinction (the first 16 trials of the three intensified groups and 16 trials of the control group). | | | | |
| --- | --- | --- | --- | --- |
| Factors | d.f., Error | *F value* | *η_p_^2^* | *p value* |
| Stimulus | 1, 113 | 213.935 | 0.654 | **< 0.001*** |
| Group | 3, 113 | 1.225 | 0.032 | 0.304 |
| Stimulus × Group | 3, 113 | 1.887 | 0.048 | 0.136 |
| * Significant results at p < 0.05. | | | | |

| **Table S.2.** Results of the mixed model ANOVAs for comparing SCR between the three intensified groups in acquisition, extinction, first and second recall phases. | | | | | |
| --- | --- | --- | --- | --- | --- |
|  | Factors | d.f., Error | *F value* | *η_p_^2^* | *p value* |
| Acquisition | Group | 2, 84 | 0.601 | 0.014 | 0.550 |
|  | Stimulus | 1, 84 | 109.625 | 0.566 | **< 0.001*** |
|  | Trial | 9.9, 835.149 | 6.524 | 0.072 | **< 0.001*** |
|  | Stimulus × Group | 2, 84 | 0.016 | 0.001 | 0.984 |
|  | Trial × Group | 19.9, 835.149 | 0.846 | 0.020 | 0.657 |
|  | Stimulus × Trial | 10.3, 861.914 | 4.985 | 0.056 | **< 0.001*** |
|  | Stimulus × Trial × Group | 20.5, 861.914 | 0.816 | 0.019 | 0.699 |
| Extinction (First 16 trials) | Group | 2, 84 | 2.593 | 0.058 | 0.081 |
|  | Stimulus | 1, 84 | 156.969 | 0.651 | **< 0.001*** |
|  | Trial | 5.1, 427.530 | 46.903 | 0.358 | **< 0.001*** |
|  | Stimulus × Group | 2, 84 | 0.414 | 0.010 | 0.662 |
|  | Trial × Group | 10.2, 427.530 | 1.063 | 0.025 | 0.390 |
|  | Stimulus × Trial | 4.3, 359.916 | 15.320 | 0.154 | **< 0.001*** |
|  | Stimulus × Trial × Group | 8.6, 359.916 | 1.777 | 0.041 | 0.075 |
| Extinction  (Second 16 trials) | Group | 2, 84 | 2.916 | 0.065 | 0.060 |
|  | Stimulus | 1, 84 | 39.409 | 0.319 | **< 0.001*** |
|  | Trial | 4.8, 399.444 | 16.988 | 0.168 | **< 0.001*** |
|  | Stimulus × Group | 2, 84 | 3.905 | 0.085 | **0.024** |
|  | Trial × Group | 9.5, 399.444 | 4.112 | 0.089 | **< 0.001*** |
|  | Stimulus × Trial | 5.5, 458.697 | 4.477 | 0.051 | **< 0.001*** |
|  | Stimulus × Trial × Group | 10.9, 458.697 | 2.392 | 0.054 | **0.007** |
| First recall | Group | 2, 84 | 0.537 | 0.544 | 0.755 |
|  | Stimulus | 1, 84 | 57.214 | 0.405 | **< 0.001*** |
|  | Trial | 5.1, 426.346 | 34.994 | 0.294 | **< 0.001*** |
|  | Stimulus × Group | 2, 84 | 0.672 | 0.016 | 0.513 |
|  | Trial × Group | 10.1, 426.346 | 0.310 | 0.007 | 0.979 |
|  | Stimulus × Trial | 5, 415.794 | 11.486 | 0.120 | **< 0.001*** |
|  | Stimulus × Trial × Group | 9.9, 415.794 | 1.118 | 0.026 | 0.347 |
| Second recall | Group | 2, 84 | 1.016 | 0.024 | 0.367 |
|  | Stimulus | 1, 84 | 26.432 | 0.239 | **< 0.001*** |
|  | Trial | 4.7, 396.116 | 19.893 | 0.191 | **< 0.001*** |
|  | Stimulus × Group | 2, 84 | 4.602 | 0.099 | **0.013*** |
|  | Trial × Group | 9.4, 396.116 | 1.086 | 0.025 | 0.372 |
|  | Stimulus × Trial | 4.5, 380.942 | 8.152 | 0.088 | **< 0.001*** |
|  | Stimulus × Trial × Group | 9.1, 380.942 | 0.801 | 0.019 | 0.617 |
| * Significant results at p < 0.05. | | | | | |

| **Table S.3.** Results of the mixed model ANOVAs for the three intensified groups: the difference between SCR generated by each CS+ and CS- presentation in each phase. | | | | | |
| --- | --- | --- | --- | --- | --- |
|  | Factors | d.f., Error | *F value* | *η_p_^2^* | *p value* |
| Acquisition | Trial | 10.3, 861.914 | 4.985 | 0.056 | **< 0.001*** |
|  | Group | 2, 84 | 0.016 | 0.001 | 0.984 |
|  | Trial × Group | 20.5, 861.914 | 0.190 | 0.019 | 0.699 |
| Extinction  (First 16 trials) | Trial | 4.3, 359.916 | 15.320 | 0.154 | **< 0.001*** |
|  | Group | 2, 84 | 0.414 | 0.010 | 0.662 |
|  | Trial × Group | 8.569, 359.916 | 1.777 | 0.041 | 0.075 |
| Extinction  (Second 16 trials) | Trial | 5.5, 458.697 | 4.777 | 0.051 | **< 0.001*** |
|  | Group | 2, 84 | 1.975 | 0.085 | **0.024*** |
|  | Trial × Group | 10.9, 458.697 | 2.392 | 0.054 | **0.007*** |
| First recall | Trial | 5, 415.794 | 11.486 | 0.120 | **< 0.001*** |
|  | Group | 2, 84 | 0.672 | 0.016 | 0.513 |
|  | Trial × Group | 9.9, 415.794 | 1.118 | 0.026 | 0.347 |
| Second recall | Trial | 4.5, 380.942 | 8.152 | 0.088 | **< 0.001*** |
|  | Group | 2, 84 | 4.602 | 0.099 | **0.013*** |
|  | Trial × Group | 9.1, 380.942 | 0.801 | 0.019 | 0.617 |
| Significant results are marked with asterisks. | | | | | |

| **Table S.4.** The PANAS values for positive and negative affects in each condition. | | | | | |
| --- | --- | --- | --- | --- | --- |
| Stimuli | Groups | Acquisition | Extinction | First recall | Second recall |
| Positive affect | a_AAA | 27.86 (7.49) | 26.54 (6.31) | 27.93 (6.53) | 27.60 (8.05) |
|  | a_ABA | 25.83 (8.91) | 25.93 (7.05) | 25.19 (6.21) | 26.17 (7.65) |
|  | a_ABB | 25.34 (7.92) | 24.68 (7.00) | 24.85 (7.07) | 26.17 (8.11) |
|  | s_AAA | 23.31 (7.55) | 24.75 (6.72) | 23.30 (6.16) | 24.57 (8.86) |
| Negative affect | a_AAA | 17.03 (6.92) | 14.96 (5.57) | 14.48 (5.00) | 18.90 (7.56) |
|  | a_ABA | 16.10 (7.28) | 15.07 (6.49) | 14.85 (5.30) | 17.83 (6.47) |
|  | a_ABB | 15.90 (6.74) | 15.04 (6.36) | 14.22 (4.77) | 16.73 (5.91) |
|  | s_AAA | 15.83 (6.43) | 14.93 (5.80) | 15.48 (6.54) | 16.53 (6.21) |
| Values are given as mean (+/- standard deviation). | | | | | |

| **Table S.5.** Absolute SCR values (µS) of each trial for different stimuli during the acquisition phase. | | | | | | | | | | | | | | | | | |
| --- | --- | --- | --- | --- | --- | --- | --- | --- | --- | --- | --- | --- | --- | --- | --- | --- | --- |
| Stimuli | Groups | Trial 1 | Trial 2 | Trial 3 | Trial 4 | Trial 5 | Trial 6 | Trial 7 | Trial 8 | Trial 9 | Trial 10 | Trial 11 | Trial 12 | Trial 13 | Trial 14 | Trial 15 | Trial 16 |
| CS+ | Massed | 0.40 (0.07) | 0.55 (0.08) | 0.39 (0.08) | 0.48 (0.08) | 0.40 (0.08) | 0.50 (0.09) | 0.43 (0.09) | 0.33 (0.07) | 0.62 (0.09) | 0.49 (0.08) | 0.50 (0.09) | 0.57 (0.09) | 0.39 (0.09) | 0.48 (0.09) | 0.36 (0.08) | 0.54 (0.10) |
|  | 20-minutes | 0.61 (0.07) | 0.59 (0.08) | 0.61 (0.08) | 0.52 (0.08) | 0.58 (0.09) | 0.60 (0.09) | 0.60 (0.09) | 0.35 (0.07) | 0.58 (0.09) | 0.40 (0.09) | 0.55 (0.09) | 0.55 (0.09) | 0.52 (0.09) | 0.46 (0.09) | 0.43 (0.08) | 0.58 (0.10) |
|  | 3-hours | 0.55 (0.07) | 0.63 (0.08) | 0.47 (0.07) | 0.51 (0.08) | 0.50 (0.08) | 0.56 (0.09) | 0.62 (0.09) | 0.48 (0.07) | 0.55 (0.09) | 0.53 (0.08) | 0.58 (0.09) | 0.55 (0.09) | 0.55 (0.08) | 0.56 (0.08) | 0.47 (0.08) | 0.50 (0.10) |
|  | Control | 0.53 (0.07) | 0.52 (0.08) | 0.50 (0.07) | 0.47 (0.08) | 0.59 (0.08) | 0.56 (0.09) | 0.42 (0.09) | 0.36 (0.07) | 0.50 (0.09) | 0.60 (0.08) | 0.50 (0.09) | 0.48 (0.09) | 0.37 (0.08) | 0.64 (0.08) | 0.40 (0.08) | 0.65 (0.10) |
| CS- | Massed | 0.42 (0.07) | 0.34 (0.07) | 0.30 (0.07) | 0.19 (0.07) | 0.22 (0.06) | 0.28 (0.06) | 0.11 (0.06) | 0.15 (0.06) | 0.13 (0.06) | 0.15 (0.05) | 0.21 (0.06) | 0.14 (0.06) | 0.17 (0.06) | 0.16 (0.05) | 0.20 (0.07) | 0.28 (0.06) |
|  | 20-minutes | 0.57 (0.07) | 0.46 (0.07) | 0.36 (0.07) | 0.32 (0.07) | 0.25 (0.06) | 0.30 (0.06) | 0.25 (0.06) | 0.27 (0.06) | 0.20 (0.06) | 0.20 (0.05) | 0.21 (0.06) | 0.27 (0.06) | 0.17 (0.06) | 0.14 (0.05) | 0.19 (0.07) | 0.25 (0.07) |
|  | 3-hours | 0.53 (0.06) | 0.46 (0.06) | 0.40 (0.07) | 0.31 (0.07) | 0.31 (0.06) | 0.24 (0.06) | 0.29 (0.06) | 0.19 (0.06) | 0.25 (0.06) | 0.23 (0.05) | 0.20 (0.06) | 0.23 (0.06) | 0.25 (0.06) | 0.20 (0.05) | 0.24 (0.07) | 0.23 (0.06) |
|  | Control | 0.49 (0.06) | 0.42 (0.06) | 0.31 (0.07) | 0.24 (0.07) | 0.16 (0.06) | 0.24 (0.06) | 0.16 (0.06) | 0.23 (0.06) | 0.15 (0.06) | 0.12 (0.05) | 0.16 (0.06) | 0.17 (0.06) | 0.14 (0.06) | 0.10 (0.05) | 0.17 (0.07) | 0.18 (0.06) |
| Values are given as mean (+/- standard deviation). | | | | | | | | | | | | | | | | | |

| **Table S.6.** Absolute SCR values (µS) of each trial for different stimuli during the extinction, first and second recall phases. | | | | | | | | | | |
| --- | --- | --- | --- | --- | --- | --- | --- | --- | --- | --- |
|  | Stimuli | Groups | Trial 1 | Trial 2 | Trial 3 | Trial 4 | Trial 5 | Trial 6 | Trial 7 | Trial 8 |
| Extinction | CS+ | Massed | 0.17 (0.09) | 0.20 (0.09) | 0.13 (0.08) | 0.18 (0.08) | 0.13 (0.07) | 0.19 (0.08) | 0.26 (0.07) | 0.16 (0.07) |
|  |  | 20-minutes | 0.82 (0.09) | 0.43 (0.09) | 0.40 (0.08) | 0.39 (0.08) | 0.32 (0.07) | 0.34 (0.08) | 0.30 (0.08) | 0.28 (0.07) |
|  |  | 3-hours | 0.66 (0.09) | 0.45 (0.08) | 0.37 (0.07) | 0.34 (0.08) | 0.19 (0.07) | 0.25 (0.08) | 0.22 (0.07) | 0.24 (0.07) |
|  |  | Control | 0.87 (0.09) | 0.75 (0.08) | 0.62 (0.07) | 0.73 (0.08) | 0.51 (0.07) | 0.46 (0.08) | 0.43 (0.07) | 0.47 (0.07) |
|  | CS- | Massed | 0.10 (0.06) | 0.18 (0.06) | 0.13 (0.05) | 0.11 (0.05) | 0.08 (0.04) | 0.05 (0.05) | 0.09 (0.05) | 0.11 (0.05) |
|  |  | 20-minutes | 0.26 (0.06) | 0.21 (0.06) | 0.17 (0.05) | 0.07 (0.05) | 0.05 (0.04) | 0.17 (0.05) | 0.16 (0.05) | 0.13 (0.05) |
|  |  | 3-hours | 0.27 (0.06) | 0.23 (0.06) | 0.13 (0.05) | 0.13 (0.05) | 0.12 (0.04) | 0.23 (0.05) | 0.08 (0.05) | 0.13 (0.05) |
|  |  | Control | 0.42 (0.06) | 0.24 (0.06) | 0.14 (0.05) | 0.17 (0.05) | 0.21 (0.04) | 0.19 (0.05) | 0.23 (0.05) | 0.28 (0.05) |
| First recall | CS+ | Massed | 0.71 (0.10) | 0.55 (0.10) | 0.35 (0.09) | 0.31 (0.09) | 0.19 (0.09) | 0.20 (0.08) | 0.18 (0.07) | 0.21 (0.09) |
|  |  | 20-minutes | 0.74 (0.11) | 0.66 (0.10) | 0.46 (0.09) | 0.48 (0.09) | 0.28 (0.09) | 0.26 (0.08) | 0.09 (0.07) | 0.25 (0.09) |
|  |  | 3-hours | 0.82 (0.10) | 0.67 (0.10) | 0.43 (0.09) | 0.44 (0.08) | 0.45 (0.08) | 0.27 (0.08) | 0.23 (0.07) | 0.26 (0.09) |
|  |  | Control | 0.67 (0.10) | 0.68 (0.10) | 0.37 (0.09) | 0.32 (0.08) | 0.31 (0.08) | 0.36 (0.08) | 0.22 (0.07) | 0.39 (0.09) |
|  | CS- | Massed | 0.33 (0.07) | 0.27 (0.06) | 0.15 (0.05) | 0.12 (0.05) | 0.14 (0.05) | 0.12 (0.05) | 0.15 (0.06) | 0.10 (0.06) |
|  |  | 20-minutes | 0.36 (0.07) | 0.20 (0.06) | 0.10 (0.05) | 0.13 (0.05) | 0.17 (0.05) | 0.08 (0.05) | 0.21 (0.06) | 0.11 (0.07) |
|  |  | 3-hours | 0.40 (0.07) | 0.25 (0.06) | 0.12 (0.05) | 0.18 (0.05) | 0.13 (0.05) | 0.23 (0.05) | 0.18 (0.06) | 0.19 (0.06) |
|  |  | Control | 0.33 (0.07) | 0.09 (0.06) | 0.23 (0.05) | 0.14 (0.05) | 0.14 (0.05) | 0.12 (0.05) | 0.15 (0.06) | 0.23 (0.06) |
| Second recall | CS+ | Massed | 0.43 (0.11) | 0.23 (0.10) | 0.14 (0.08) | 0.05 (0.06) | 0.13 (0.07) | 0.05 (0.06) | 0.09 (0.06) | 0.08 (0.07) |
|  |  | 20-minutes | 0.65 (0.11) | 0.54 (0.10) | 0.28 (0.08) | 0.31 (0.07) | 0.22 (0.07) | 0.20 (0.06) | 0.22 (0.07) | 0.14 (0.07) |
|  |  | 3-hours | 0.55 (0.11) | 0.39 (0.10) | 0.27 (0.08) | 0.18 (0.06) | 0.21 (0.07) | 0.15 (0.06) | 0.14 (0.06) | 0.17 (0.06) |
|  |  | Control | 0.49 (0.11) | 0.32 (0.10) | 0.31 (0.08) | 0.26 (0.06) | 0.20 (0.07) | 0.14 (0.06) | 0.22 (0.06) | 0.26 (0.06) |
|  | CS- | Massed | 0.19 (0.06) | 0.12 (0.05) | 0.11 (0.06) | 0.12 (0.05) | 0.13 (0.06) | 0.13 (0.04) | 0.06 (0.05) | 0.14 (0.07) |
|  |  | 20-minutes | 0.23 (0.06) | 0.24 (0.05) | 0.12 (0.06) | 0.09 (0.05) | 0.14 (0.06) | 0.06 (0.04) | 0.13 (0.05) | 0.16 (0.07) |
|  |  | 3-hours | 0.27 (0.05) | 0.05 (0.05) | 0.21 (0.06) | 0.12 (0.05) | 0.11 (0.05) | 0.07 (0.04) | 0.20 (0.05) | 0.15 (0.07) |
|  |  | Control | 0.15 (0.05) | 0.17 (0.05) | 0.05 (0.06) | 0.11 (0.05) | 0.13 (0.05) | 0.14 (0.04) | 0.12 (0.05) | 0.18 (0.07) |
| Values are given as mean (+/- standard deviation). | | | | | | | | | | |

| **Table S.7.** Differential SCR values (CS+ - CS-) for each trial in the four phases. | | | | | | | | | | | | | | | | | |
| --- | --- | --- | --- | --- | --- | --- | --- | --- | --- | --- | --- | --- | --- | --- | --- | --- | --- |
| Stimuli | Groups | Trial 1 | Trial 2 | Trial 3 | Trial 4 | Trial 5 | Trial 6 | Trial 7 | Trial 8 | Trial 9 | Trial 10 | Trial 11 | Trial 12 | Trial 13 | Trial 14 | Trial 15 | Trial 16 |
| Acquisition | Massed | -0.03 (0.43) | 0.21 (0.36) | 0.09 (0.32) | 0.29 (0.36) | 0.18 (0.36) | 0.22 (0.56) | 0.32 (0.49) | 0.18 (0.35) | 0.49 (0.52) | 0.34 (0.39) | 0.29 (0.51) | 0.43 (0.51) | 0.21 (0.52) | 0.32 (0.49) | 0.15 (0.35) | 0.26 (0.47) |
|  | 20-minutes | 0.04 (0.27) | 0.13 (0.37) | 0.25 (0.38) | 0.19 (0.36) | 0.32 (0.44) | 0.31 (0.48) | 0.35 (0.44) | 0.09 (0.47) | 0.39 (0.48) | 0.20 (0.43) | 0.34 (0.59) | 0.28 (0.47) | 0.35 (0.59) | 0.32 (0.49) | 0.24 (0.33) | 0.33 (0.54) |
|  | 3-hours | 0.02 (0.32) | 0.17 (0.42) | 0.07 (0.34) | 0.20 (0.47) | 0.19 (0.35) | 0.32 (0.49) | 0.33 (0.46) | 0.30 (0.42) | 0.29 (0.57) | 0.30 (0.48) | 0.38 (0.39) | 0.32 (0.44) | 0.30 (0.62) | 0.36 (0.45) | 0.22 (0.39) | 0.27 (0.36) |
|  | Control | 0.03 (0.36) | 0.10 (0.34) | 0.19 (0.39) | 0.23 (0.30) | 0.43 (0.42) | 0.33 (0.39) | 0.26 (0.39) | 0.13 (0.31) | 0.35 (0.50) | 0.48 (0.42) | 0.34 (0.50) | 0.32 (0.44) | 0.23 (0.38) | 0.53 (0.49) | 0.23 (0.31) | 0.46 (0.50) |
| Extinction | Massed | 0.07 (0.24) | 0.02 (0.27) | 0.00 (0.25) | 0.07 (0.30) | 0.05 (0.23) | 0.14 (0.27) | 0.17 (0.46) | 0.06 (0.33) | NA | NA | NA | NA | NA | NA | NA | NA |
|  | 20-minutes | 0.56 (0.59) | 0.22 (0.60) | 0.23 (0.41) | 0.32 (0.50) | 0.27 (0.46) | 0.17 (0.50) | 0.14 (0.46) | 0.15 (0.46) | NA | NA | NA | NA | NA | NA | NA | NA |
|  | 3-hours | 0.40 (0.47) | 0.22 (0.36) | 0.24 (0.41) | 0.21 (0.48) | 0.07 (0.22) | 0.02 (0.31) | 0.14 (0.28) | 0.11 (0.40) | NA | NA | NA | NA | NA | NA | NA | NA |
|  | Control | 0.45 (0.44) | 0.50 (0.53) | 0.48 (0.46) | 0.56 (0.51) | 0.31 (0.43) | 0.27 (0.44) | 0.20 (0.46) | 0.19 (0.45) | NA | NA | NA | NA | NA | NA | NA | NA |
| First recall | Massed | 0.37 (0.46) | 0.29 (0.53) | 0.20 (0.39) | 0.19 (0.32) | 0.06 (0.23) | 0.08 (0.33) | 0.03 (0.41) | 0.11 (0.44) | NA | NA | NA | NA | NA | NA | NA | NA |
|  | 20-minutes | 0.38 (0.49) | 0.47 (0.51) | 0.36 (0.44) | 0.35 (0.48) | 0.10 (0.30) | 0.18 (0.43) | -0.12 (0.46) | 0.14 (0.45) | NA | NA | NA | NA | NA | NA | NA | NA |
|  | 3-hours | 0.42 (0.50) | 0.42 (0.45) | 0.31 (0.60) | 0.26 (0.46) | 0.32 (0.50) | 0.04 (0.40) | 0.05 (0.56) | 0.07 (0.64) | NA | NA | NA | NA | NA | NA | NA | NA |
|  | Control | 0.34 (0.51) | 0.59 (0.50) | 0.15 (0.48) | 0.18 (0.39) | 0.17 (0.46) | 0.23 (0.32) | 0.08 (0.29) | 0.16 (0.54) | NA | NA | NA | NA | NA | NA | NA | NA |
| Second recall | Massed | 0.23 (0.44) | 0.11 (0.37) | 0.03 (0.28) | -0.07 (0.30) | 0.00 (0.34) | -0.08 (0.22) | 0.03 (0.16) | -0.06 (0.26) | NA | NA | NA | NA | NA | NA | NA | NA |
|  | 20-minutes | 0.42 (0.60) | 0.31 (0.60) | 0.16 (0.47) | 0.22 (0.40) | 0.08 (0.27) | 0.14 (0.44) | 0.09 (0.34) | -0.02 (0.42) | NA | NA | NA | NA | NA | NA | NA | NA |
|  | 3-hours | 0.28 (0.52) | 0.34 (0.55) | 0.06 (0.37) | 0.06 (0.18) | 0.10 (0.39) | 0.08 (0.36) | -0.06 (0.33) | 0.02 (0.52) | NA | NA | NA | NA | NA | NA | NA | NA |
|  | Control | 0.34 (0.55) | 0.15 (0.47) | 0.27 (0.45) | 0.15 (0.47) | 0.07 (0.58) | -0.01 (0.36) | 0.10 (0.43) | 0.08 (0.59) | NA | NA | NA | NA | NA | NA | NA | NA |
| Values are given as mean (+/- standard deviation). “NA” means not applicable. | | | | | | | | | | | | | | | | | |

| **Table S.8.** Valence, arousal and fear ratings for each stimulus in different conditions for each phase. | | | | | | |
| --- | --- | --- | --- | --- | --- | --- |
|  | Groups | Stimuli | Acquisition | Extinction | First recall | Second recall |
| Valence | Massed | CS+ | 4.00 (0.11) | 2.93 (0.15) | 2.59 (0.17) | 2.52 (0.17) |
|  |  | CS- | 1.76 (0.15) | 1.62 (0.16) | 1.41 (0.14) | 1.48 (0.14) |
|  | 20-minutes | CS+ | 3.96 (0.12) | 3.21 (0.15) | 2.82 (0.17) | 2.18 (0.18) |
|  |  | CS- | 1.86 (0.15) | 1.54 (0.16) | 1.82 (0.15) | 1.61 (0.14) |
|  | 3-hours | CS+ | 3.93 (0.11) | 3.17 (0.15) | 2.73 (0.16) | 2.63 (0.17) |
|  |  | CS- | 1.80 (0.14) | 1.43 (0.15) | 1.47 (0.14) | 1.57 (0.14) |
|  | Control | CS+ | 3.93 (0.11) | 3.33 (0.15) | 2.80 (0.16) | 2.20 (0.17) |
|  |  | CS- | 1.77 (0.14) | 1.60 (0.15) | 1.47 (0.14) | 1.23 (0.14) |
| Arousal | Massed | CS+ | 0.64 (0.05) | 0.44 (0.04) | 0.28 (0.05) | 0.24 (0.04) |
|  |  | CS- | 0.12 (0.03) | 0.09 (0.03) | 0.05 (0.02) | 0.03 (0.02) |
|  | 20-minutes | CS+ | 0.65 (0.05) | 0.57 (0.04) | 0.30 (0.05) | 0.15 (0.04) |
|  |  | CS- | 0.17 (0.03) | 0.14 (0.03) | 0.06 (0.02) | 0.03 (0.02) |
|  | 3-hours | CS+ | 0.61 (0.05) | 0.53 (0.04) | 0.33 (0.05) | 0.28 (0.04) |
|  |  | CS- | 0.16 (0.03) | 0.08 (0.03) | 0.06 (0.02) | 0.05 (0.01) |
|  | Control | CS+ | 0.65 (0.05) | 0.49 (0.04) | 0.40 (0.05) | 0.23 (0.04) |
|  |  | CS- | 0.16 (0.03) | 0.13 (0.03) | 0.11 (0.02) | 0.05 (0.01) |
| Fear | Massed | CS+ | 0.63 (0.05) | 0.36 (0.05) | 0.29 (0.05) | 0.21 (0.04) |
|  |  | CS- | 0.10 (0.03) | 0.05 (0.03) | 0.03 (0.02) | 0.01 (0.01) |
|  | 20-minutes | CS+ | 0.51 (0.05) | 0.48 (0.05) | 0.28 (0.05) | 0.12 (0.04) |
|  |  | CS- | 0.12 (0.03) | 0.08 (0.03) | 0.06 (0.02) | 0.02 (0.01) |
|  | 3-hours | CS+ | 0.65 (0.04) | 0.51 (0.05) | 0.28 (0.05) | 0.26 (0.04) |
|  |  | CS- | 0.11 (0.03) | 0.08 (0.03) | 0.03 (0.02) | 0.02 (0.01) |
|  | Control | CS+ | 0.64 (0.04) | 0.51 (0.05) | 0.36 (0.05) | 0.21 (0.04) |
|  |  | CS- | 0.13 (0.03) | 0.14 (0.03) | 0.09 (0.02) | 0.04 (0.01) |
| Values are given as mean (+/- standard deviation). | | | | | | |

**
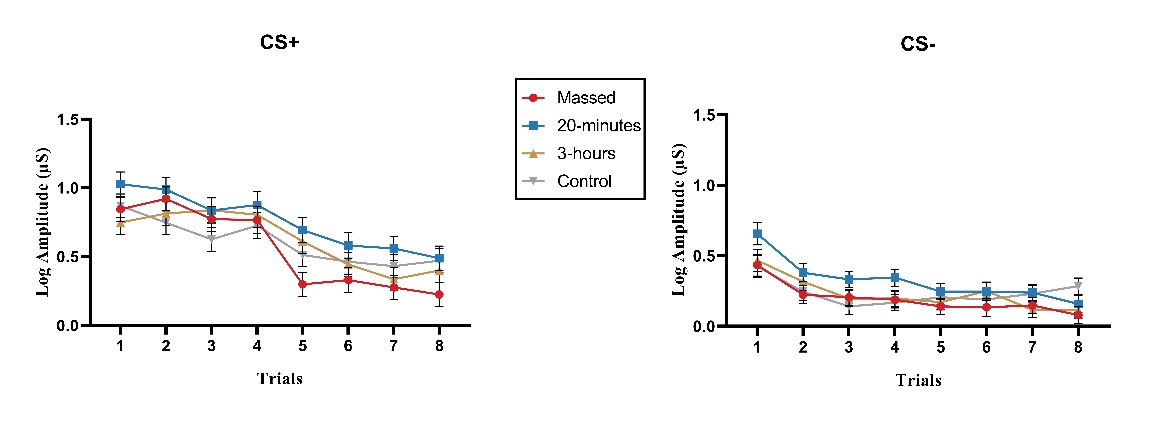
**

**Fig S.1.** The SCR in response to the CS+ and CS- for the first 16 trials of the three intensified groups and the 16 trials of the control group in the extinction phase is shown. The line color indicates the different extinction protocols, and error bars refer to the standard error of mean.

**Figure S.2.** The scores of valence, arousal and fear ratings for each study phase, shown separately for CS+ and CS-. The color of the bars shows the stimulus for each phase, and error bars show the standard error of the mean.
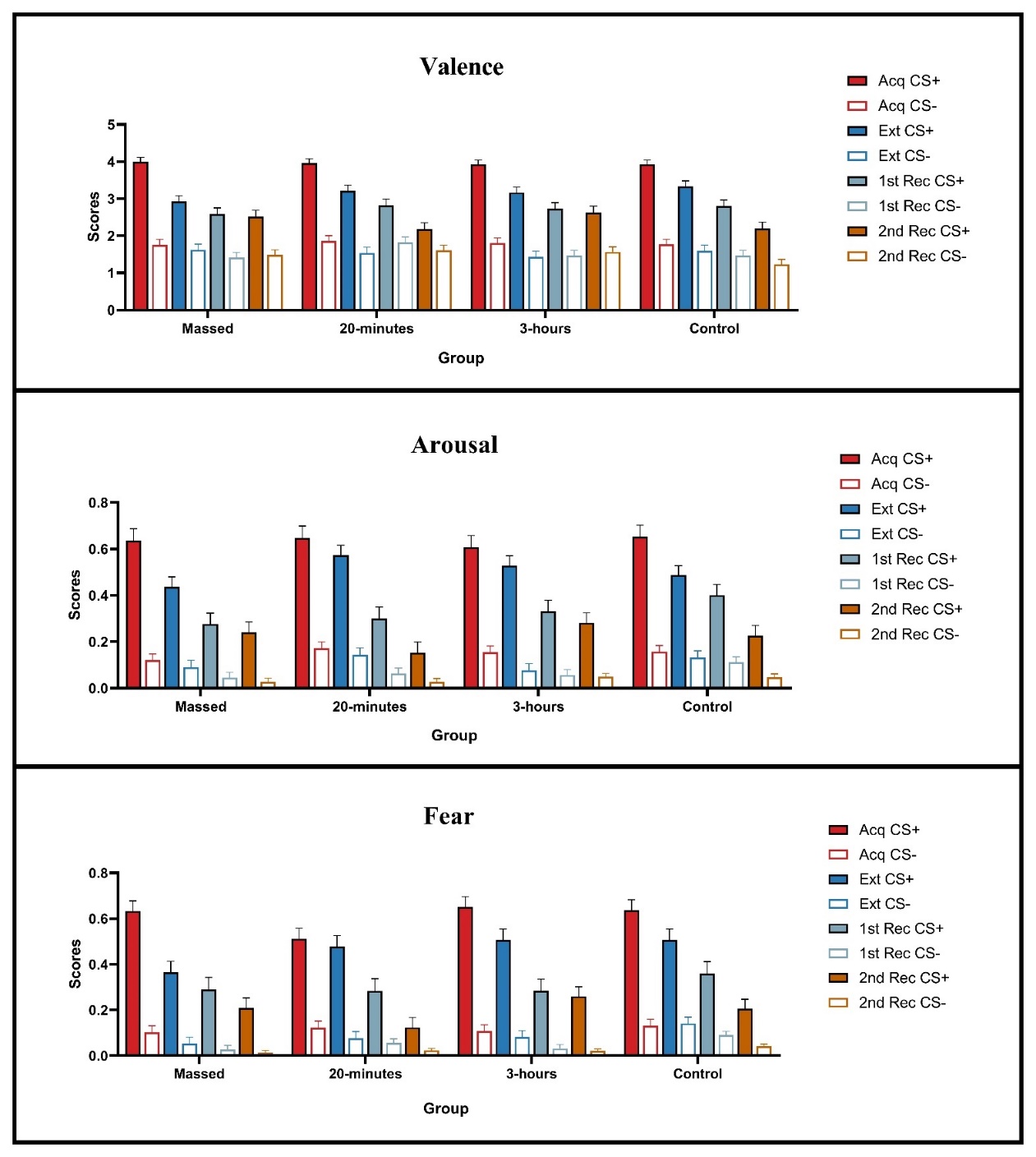

Supplement: Supplementary file 1 [file mmc1.docx]
